# SaNAR2.2, A Novel Member of the NAR2 Family from the Euhalophyte *Suaeda altissima*: Molecular Cloning and Characterization

**DOI:** 10.3390/membranes16080267

**Published:** 2026-08-12

**Authors:** Dmitry E. Khramov, Olga I. Nedelyaeva, Anastasia Ryabova, Vadim Volkov, Larissa G. Popova

**Affiliations:** 1 K.A. Timiryazev Institute of Plant Physiology, Russian Academy of Sciences, Moscow 127276, Russia; khramov.de@yandex.ru (D.E.K.); olga.nedelyaeva@yandex.ru (O.I.N.); 2 Prokhorov General Physics Institute, Russian Academy of Sciences, Moscow 119991, Russia; nastya.ryabova@gmail.com

**Keywords:** anion transporter, euhalophyte, NAR2 family, nitrate transport, *Suaeda altissima*

## Abstract

NAR2 family proteins are involved in the uptake of nitrate by plant roots as partners of high-affinity nitrate transporters of NRT2 family. In this study, we cloned the coding sequence of a gene, which encodes a protein consisting of 196 amino acids with a calculated molecular weight of 21.7 kDa, from the euhalophyte *Suaeda altissima*. The protein was characterized in terms of structure and phylogeny. On the phylogenetic tree of NAR2 family proteins, the protein from *S. altissima* is located in a clade with proteins from the NAR2.2 subfamily found in species within the Amaranthaceae. Therefore, on the basis of phylogenetic relationships, we named it SaNAR2.2. According to a prediction of cellular localization and protein topology, SaNAR2.2 is a likely resident of the plasma membrane. We detected the interaction between SaNAR2.2 and putative partner proteins SaNRT2.1 and SaNRT2.5, which are members of the high-affinity nitrate transporter family NRT2 in *S. altissima*, using the bimolecular fluorescence complementation (BiFC) assay in *Nicotiana benthamiana* leaves and the yeast mating-based split-ubiquitin system (mbSUS). *SaNAR2.2* was expressed predominantly in roots of *S. altissima*. The level of *SaNAR2.2* transcripts significantly increases at low nitrate concentrations in the medium at the background of high NaCl concentrations (up to 0.5 M). These findings point to the important role of SaNAR2.2 as a participant in the high-affinity nitrate transport system under saline conditions.

## 1. Introduction

Nitrogen is one of the essential biogenic elements. Most often, plants obtain nitrogen as a nitrate (NO_3_^−^), which is the dominant form among nitrogen compounds in aerobic soils [1]. Plants absorb nitrate from the soil solution using specialized transport proteins that function in the plasma membranes of root cells. Currently, the proteins of the NRT1/PTR/NPF (Nitrate Transporter 1/Peptide Transporter Family/Nitrate Peptide Family) and NRT2 (Nitrate Transporter 2) families are identified as significantly involved in nitrate uptake by plant roots [2,3,4]. Low-affinity transporters belonging to the NRT1/PTR/NPF family make a major contribution to nitrate uptake at high external nitrate concentrations, above 1 mM [5,6]. At low nitrate concentrations (from 1 µM to 0.5 mM) in the environment, high-affinity nitrate transporters of the NRT2 family play a decisive role in plant nitrogen nutrition [7,8].

The essential feature of NRT2 proteins is their capability to transfer NO_3_^−^ across the membrane when forming a complex with a partner protein from the NAR2/NRT3 family (Nitrate Assimilation Related Protein 2/Nitrate Transporter 3) [9,10]. NAR2 is a family of small membrane-associated proteins with molecular masses of 25–30 kD that are important components of the high-affinity nitrate uptake transport system (HATS) in plants [5,11]. Genes belonging to the *NAR2* family have been identified in a number of plant species: three genes (*HvNAR2.1*–*HvNAR2.3*) have been found in barley (*Hordeum vulgare*) [12], two genes were found in both *Arabidopsis thaliana* (*AtNAR2.1* and *AtNAR2.2*) [13] and rice (*Oryza sativa*, *OsNAR2.1* and *OsNAR2.2*) [14], and one *NAR2* gene was revealed in each of chrysanthemum (*Chrysanthemum morifolium*) [15], maize (*Zea mays*) [16,17], seagrass *Zostera marina* [18], wheat (*Triticum aestivum*) [19], and spinach (*Spinacia oleracea*) [20].

In a series of experiments, it has been shown that in higher plants, the high-affinity nitrate uptake system requires a functional NAR2 gene. In the *Arabidopsis atnar2.1* mutant, there was an almost complete loss of nitrate absorption by the roots in the presence of low nitrate concentrations in the medium (from 10 µM to 150 µM), indicating the important role of the *AtNAR2.1* gene product as a regulator of HATS activity [13,21]. Experiments in which *AtNRT2.1* and/or *AtNRT3.1*/*AtNAR2.1* mRNA were injected into *Xenopus* oocytes, either individually or together, showed that significant absorption of ^15^NO_3_^−^ in oocytes was only noticeable after the combined injection of both genes [13]. Functional two-component nitrate transport was reconstituted in *Xenopus* oocytes using the barley genes *HvNRT2.1* and *HvNAR2.3* [12].

There are numerous examples demonstrating that the NRT2 and NAR2 proteins directly interact with each other, and this interaction is localized in the plasma membrane [9,10,13]. In *A. thaliana*, two members of the NAR2 family, AtNAR2.1 and AtNAR2.2, have been identified [13]. Using the bimolecular fluorescence complementation assay and membrane yeast split-ubiquitin system, it has been shown that, with the exception of AtNRT2.7, all other members of the high-affinity NRT2 family of *A. thaliana* interact with AtNAR2.1 [10]. Also in experiments with *A. thaliana*, data indicated that the active transport form of the NRT2/NAR2 protein complex was likely a tetramer located in the plasma membrane; the complex presumably consists of two subunits, each of them a heterodimer of NRT2.1 and NAR2.1 [9,22]. The involvement of NAR2 proteins in nitrate transport as direct partners of NRT2 proteins was proved for other plant species, including rice [14], spinach [20], barley [23], wild soybean (*Glycine soja*) [24], rapeseed (*Brassica napus*) [25], and the seagrass *Z. marina* [26].

The information available on plant nitrate transporters has been obtained mainly from salt-sensitive glycophyte species, particularly from the model plant *A. thaliana*. Data about nitrate transporters in salt-tolerant halophytic plants are very limited. Currently, only one example of of a high-affinity transport system NRT2/NAR2 has been described in a marine halophyte, seagrass *Zostera marina*, namely, ZosmaNRT2/ZosmaNAR2 [18,26]. However, during soil salinization, a more complex situation is observed when sodium chloride reduces the availability of nitrate for land plants, possibly because of direct competition between the anions NO_3_^−^ and Cl^−^ for high-affinity nitrate transporters [27,28]. Under these conditions, NO_3_^−^ uptake and nitrogen assimilation in glycophytes are suppressed [29,30]. In highly salt-tolerant halophytes growing on saline substrates, nitrate-transporting plasma membrane proteins are able to function while in contact with an external solution with a high chloride concentration. This suggests the presence of structural features in halophyte proteins that allow them to bind nitrate and transport it across the membrane when the concentration of this anion in the surrounding solution is much lower than the concentration of chloride ions. It can also be predicted that under conditions of nitrate deficiency and simultaneous salinization of the nutrient medium with sodium chloride, high-affinity nitrate transporters play a key role in the absorption of nitrate ions by roots.

Many representatives of the genus *Suaeda* (Amaranthaceae/Chenopodiaceae) have been identified as highly salt-tolerant species [31,32,33,34,35]. *Suaeda altissima* is one of the most salt-tolerant plant species, able to proliferate at high NaCl concentrations in the nutrient medium (up to 750 mM). Moreover, growth of *S. altissima* is not reduced at a NaCl concentration of around 250 mM, but stimulated [34]. It should be stressed that the concentration of 250 mM NaCl is lethal for glycophytes [36]. At high NaCl concentrations, chloride likely competes with nitrate for binding sites on nitrate transporters. Halophytes, including *S. altissima*, may successfully complete their life cycle in highly saline and nitrate-poor soils due to the unique characteristics of their high-affinity nitrate transporters, which facilitate nitrate uptake despite considerable competition from chloride.

In our previous research, we cloned the coding regions of two genes from the euhalophyte *S. altissima*, *SaNRT2.1* (OR909030.1) and *SaNRT2.5* (OR828748.1), which belong to the high-affinity nitrate transporter gene family *NRT2* [37]. The proteins SaNRT2.1 and SaNRT2.5 from *S. altissima* are orthologs of high-affinity nitrate transporters AtNRT2.1 and AtNRT2.5 from *A. thaliana*, respectively, which were functionally active only in complex with the protein AtNAR2.1/AtNRT3.1 [10]. It is assumed that SaNRT2.1 and SaNRT2.5 also form functional complexes with proteins of the NAR2/NRT3 family. The present work is aimed at the identification of the coding sequence of the *NAR2* family gene in *S. altissima*, *SaNAR2.2*. We studied the expression of this gene in organs of *S. altissima* at different concentrations of nitrate and chloride in the nutrient medium. Using the bimolecular fluorescence complementation (BiFC) method [38,39] and the yeast mating-based split-ubiquitin system (mbSUS) [40], we demonstrated that protein SaNAR2.2 associates with the proteins SaNRT2.1 and SaNRT2.5. To analyze the function of the SaNAR2.2 protein and NRT2 family transporters from *S. altissima*, the coding sequences *SaNAR2.2* and *SaNRT2.1/SaNRT2.5* were co-expressed in the *Δynt1* mutant strain of *Hansenula (Ogatae) polymorpha* yeast. Methylotrophic yeast *H. polymorpha* is able to use nitrate as the only nitrogen source and possesses the genes required for its uptake from the medium and assimilation [41]. The high-affinity nitrate uptake in *H. polymorpha* is mediated by a single transporter encoded by the *YNT1* gene (Yeast Nitrate Transporter 1) [42]. The knockout mutant *H. polymorpha*, *Δynt1*, is used to study heterologously expressed nitrate transporters from plants [41,43,44].

## 2. Materials and Methods

### 2.1. Plant Material

The euhalophyte *Suaeda altissima* (L.) Pall. was the object of the study for this research. *S. altissima* seeds collected from plants growing in the vicinity of the salt lake Elton (Volgograd Region, Russia), were germinated in wet sand. Seedlings aged 14 days were transferred to 3 L vessels with liquid nutrient medium (four plants per vessel). The plants were grown in hydroponic culture on aerated Robinson–Downton nutrient medium [45]. The plants were grown at two concentrations of NO_3_^−^ in the medium, low (0.5 mM) and high (15 mM), and four concentrations of Cl^−^ (0, 250, 500, and 750 mM) for each of the specified nitrate concentrations. Nitrate and chloride were added to the nutrient solution in the form of KNO_3_ and NaCl, respectively. NaCl was added to the nutrient solution starting from the seventh day after the transfer of seedlings to the hydroponic culture. The concentration of NaCl in the nutrient solution gradually increased by 50–100 mM per day until the final concentrations of 250, 500, and 750 mM were reached. The nutrient medium for the control plants did not include NaCl. Seed germination and plant cultivation were carried out at 24 °C and a relative humidity of 60–70%. The plants were illuminated with high-pressure sodium lamps DNaZ_250 (Reflux, Saransk, Russia) (300 µmol photons m^−2^ s^−1^, photoperiod of 16 h light/8 h dark).

Tobacco *Nicotiana benthamiana* Domin was used as an auxiliary plant species. The plants of *N. benthamiana* were grown from seeds in a soil substrate at 23 ± 2 °C, relative air humidity of 70 ± 5%, photoperiod of 16 h light/8 h dark, and light intensity of 120 µmol quanta/(m^2^·s) emitted by Philips TL-D-58W/33-640 fluorescent lamps (Philips, Amsterdam, The Netherlands). For transient expression of *S. altissima* genes in *N. benthamiana*, forty-day-old *N. benthamiana* plants were used.

### 2.2. Isolation of Total RNA from Plant Material and cDNA Synthesis

To obtain total RNA from *S. altissima* organs, 6-weeks-old plants were used. Samples weighing 0.5 g of plant material (roots, leaves, stems) were used for RNA extraction. Total RNA from *S. altissima* organs was extracted by the hot phenol method as described in [46] and treated with DNase I (Thermo Fisher Scientific, Inc., Waltham, MA, USA) to remove genomic DNA contaminants. The concentration and purity of the RNA samples were determined spectrophotometrically by the A260/A280 ratio using the NanoDrop ND1000 device (Thermo Fisher Scientific, Inc., Waltham, MA, USA). One µg aliquots of DNase-treated total RNA in a total volume of 20 µL were used as templates for the synthesis of the first-strand cDNA.

### 2.3. Primer Design

Primers for quantitative real-time PCR (qRT-PCR) were designed using Primer-BLAST v4.1.0 (https://www.ncbi.nlm.nih.gov/tools/primer-blast/ (accessed on 2 November 2024)). Primers for all other applications were designed using SnapGene Viewer v7.2 (https://www.snapgene.com/ (accessed on 2 November 2024)). The sequences of all primers used in this study are listed in the Supplementary Table S1.

### 2.4. Cloning of the Full-Length Coding Sequence SaNAR2.2

To clone the full-length coding sequence of the *NAR2* gene from *S. altissima*, the sequences of its 5′- and 3′-end fragments were initially determined using the Step-Out RACE method (kit No. SKS03, Evrogen, Moscow, Russia). The amplification of the 5′- and 3′-end fragments of the targeted *NAR2* transcript, as well as the subsequent amplification of the full-length coding sequence of *NAR2* from *S. altissima*, was carried out on the first-strand cDNA template synthesized in a reverse transcription reaction on a total RNA template isolated from the roots of *S. altissima*. In these experiments, the first-strand cDNA was synthesized using MINT reverse transcriptase (Evrogen, Moscow, Russia) and the (dT)_12_VN primer.

For the amplification of the 5′- and 3′-end fragments, we used the Encyclo DNA polymerase (Cat. No. PK002, Evrogen, Moscow, Russia) and gene-specific primers designed based on the previously identified partial coding sequence of the *NAR2* family gene from *S. altissima* [47]. The 5′-end of *SaNAR2* cDNA was amplified with primers SaNRT3.2_R (round 1) and SaNRT3.2_R1 (round 2). The 3′-end of *SaNAR2* cDNA was amplified with SaNRT3.2_F (round 1) and SaNRT3.2_F1 (round 2). The primers Mix1 (round 1) and Mix2 (round 2) from the manufacturer’s kit were also used for amplification of *SaNAR2* fragments. Using the Quick-TA kit (Cat. No. TAK02, Evrogen, Moscow, Russia), the amplicons obtained were cloned into the pAL2-T vector for replication in *Escherichia coli* cells (strain XL1 Blue) and sequenced.

Overlapping sequences of the 5′- and 3′-ends of the *SaNAR2* coding sequence were in silico aligned with each other using SnapGene Viewer software, v. 5.0.8 (https://www.snapgene.com/snapgene-viewer (accessed on 2 November 2024)). The resulting sequence obtained in this way contained an open reading frame of 591 bp. Based on this sequence, the gene-specific primers SaNRT3.2_F3 and SaNRT3.2_R3 were selected, complementary to the 5′- and 3′- ends of the full-length coding sequence *SaNAR2*. The full-length cDNA of *SaNAR2* was amplified on the first-strand cDNA template using these primers and the Encyclo DNA polymerase (Cat. No. PK002, Evrogen, Moscow, Russia) for the first 15 cycles, and the ready-to-use CloneAmp HiFi PCR Premix (Cat. No. 639298, Clontech, Mountain View, CA, USA) for the next 30 cycles. An amplicon of 591 bp was obtained, named *SaNAR2.2* (see Results Section), which was further cloned into the yeast integrative vector pCCUR2 under the control of the inducible nitrate reductase (YNR1) promoter *pYNR1* and terminator *tYNR1* of yeast *Hansenula polymorpha*. To merge the *SaNAR2.2* sequence with the *pYNR1* and *tYNR1*, we used the previously obtained construct pCHLX-pYNR1-SaNPF6.3-tYNR1 [44], which already contained the *pYNR1* and *tYNR1* sequences. The construct pCHLX-pYNR1-SaNPF6.3-tYNR1 was linearized by the inverse PCR method with primers pYNR1-SaNRT3.2-R and tYNR1-SaNRT3.2-F using the CloneAmp HiFi PCR Premix kit, after which the linearized vector pCHLX flanked with *pYNR1* and *tYNR1* sequences was ligated with the *SaNAR2.2* sequence using the Gibson assembly kit (Cat. No. E5510, NEB, Ipswich, MA, USA). Subsequently, the obtained pCHLX-pYNR1-SaNAR2.2-tYNR1 construct was used as a template for amplifying the *pYNR1-SaNAR2.2-tYNR1* sequence with primers pCCUR2-pYNR1-F and pCCUR2-tYNR1-R, followed by the subcloning of this fragment into the pCCUR2 vector. The vector pCCUR2 was linearized at the KpnI restriction site and ligated with the *pYNR1-SaNAR2.2-tYNR1* fragment using the Gibson assembly kit to produce the pCCUR2-pYNR1-SaNAR2.2-tYNR1 construct (further denoted as pCCUR2-SaNAR2.2) (Figures S1–S4).

The bacterial *E. coli* XL1 Blue strain was used for routine plasmid propagation. The cloned sequence *SaNAR2.2* (591 bp) was verified by sequencing (Evrogen, Moscow, Russia).

### 2.5. Quantitative Analysis of SaNAR2.2 Transcripts in S. altissima Organs

Quantitative analysis of *SaNAR2.2* transcripts in *S. altissima* organs was performed by quantitative real-time PCR (qRT-PCR) using the LightCycler^®^ 96 System (Roche Diagnostics Corporation, Indianapolis, IN, USA). The cDNA samples for the analysis of *SaNAR2.2* expression were synthesized on templates of the total RNA isolated from the organs (roots, leaves, stems) of *S. altissima* plants, grown at various concentrations of nitrate and NaCl in the medium.

PCR analysis was performed using a ready-to-use reaction mixture containing the intercalating dye SYBR Green I (Evrogen, Moscow, Russia). As a reference gene, the elongation factor 1 alpha gene from *S. altissima*, *SaeEF1alpha* (GenBank ID: MN076325.1), was used. Earlier, we showed that the expression of this housekeeping gene was constitutive in various *S. altissima* organs and under different experimental conditions [48]. The PCR reactions (in triplicate) included 50 ng of cDNA and 0.5 µM of each primer in a total volume of 20 μL. The reactions were initially denatured (95 °C/300 s), then subjected to 45 cycles of 95 °C/20 s, 54 °C/15 s, 72 °C/15 s. The results were processed by LightCycler 96SW 1.1 software. The results obtained in these experiments are based on three biological replicates. Primer specificity for each gene was evaluated by analyzing the melting curves of PCR products. The relative transcript abundance was calculated by the 2^−∆∆CT^ method.

### 2.6. Bimolecular Fluorescence Complementation (BiFC) in Nicotiana benthamiana Leaves

Plasmid vector constructs for analyzing the interaction of the SaNAR2.2 protein with NRT2 family proteins from *S. altissima*, SaNRT2.1 and SaNRT2.5 were obtained based on the pSPYNE-35S and pSPYCE-35S plasmids [49,50]. The plasmids contain, respectively, the N-terminal fragment (155 aa) and the C-terminal fragment (84 aa) of the fluorescent protein mVenus under the control of the constitutive cauliflower mosaic virus 35S promoter. The sequence *SaNAR2.2* was subcloned into the pSPYCE-35S plasmid upstream of the C-terminal fragment of mVenus, and the resulting construct was designated as pSPYCE-35S-SaNAR2.2. The sequences *SaNRT2.1* (OR909030.1) or *SaNRT2.*5 (OR828748.1) encoding high-affinity nitrate transporters of the NRT2 family from *S. altissima* were cloned into the pSPYNE-35S plasmid upstream of the sequence encoding the N-terminal fragment of mVenus. Accordingly, the constructs pSPYNE-35S-SaNRT2.1 and pSPYNE-35S-SaNRT2.5 were obtained. The constructs obtained were transferred into *Agrobacterium tumefaciens*, strain GV3101. The transformation of bacteria was carried out using the freeze–thaw method [51]. The transformed bacteria were used for the transient transformation of epidermal cells of *N. benthamiana* leaves. Bacterial transformants, as well as the helper strain *A. tumefaciens* containing the coding sequence for the protein p19, were induced with acetosyringone for 3 h [52]. Suspensions of transformants carrying plasmids with transgenes from *S. altissima* were adjusted to 0.7 OD_600_, while the suspension of the helper strain was adjusted to 1.0 OD_600_. Suspensions of the helper strain, the transformant carrying *SaNAR2.2*, and the transformant carrying *SaNRT2.1/SaNRT2.5* were mixed in equal volumes, and the resulting mixture was used to infiltrate the leaves of forty-day-old *N. benthamiana* plants on the abaxial side. After infiltration, the plants were grown at 21 °C for 3 days for transgene expression, after which the leaf fragments were examined with a laser scanning confocal microscope LSM-710-NLO (Carl Zeiss, Jena, Germany). In these experiments, a low-affinity nitrate transporter gene *SaNPF6.3 (*GenBank ID: OQ330855.1*)* of the *NPF* family from *S. altissima* [44] was co-expressed with *SaNAR2.2* in the leaves of *N. benthamiana* as a negative control, for which the construct pSPYNE-35S-SaNPF6.3 was obtained.

The images of the fluorescence signal from mVenus in *N. benthamiana* cells transformed with the vector constructs were obtained at λ_ex_ 515 nm and λ_em_ 527 nm. The transmitted laser light was recorded with a separate T-PMT detector.

### 2.7. Yeast Mating-Based Split-Ubiquitin Assay (mbSUS)

To investigate potential interaction between the proteins SaNAR2.2 and SaNRT2.1/SaNRT2.5, the yeast mating-based split-ubiquitin system (mbSUS) was also used [40].

In these experiments, the following *Saccharomyces cerevisiae* haploid strains were used: the strain THY.AP4 (genotype: MATα; ade2^−^, his3^−^, leu2^−^, trp1^−^, ura3^−^; lexA::ADE2, lexA::HIS3, lexA::lacZ) and the strain THY.AP5 (MATα; URA3; ade2^−^, his3^−^, leu2^−^, trp1^−^). Vectors pMetYC-Dest for bait and pNX35-Dest for prey were used to co-express the studied proteins, SaNAR2.2 + SaNRT2.1 or SaNAR2.2 + SaNRT2.5, in the diploid yeast strain THY.AP4/THY.AP5.

Vector pNX35-Dest was linearized at the AatII restriction sites flanking the ccdB sequence. Vector pMetYC-Dest was linearized by inverse PCR with the high-fidelity polymerase CloneAmp HiFi Polymerase (CloneAmp HiFi PCR Premix, cat. No. 639298, Clontech, Takara, San Jose, CA, USA) and primers pMetYC-R and pMetYC-F (Supplementary Table S1). The coding sequences of the *SaNAR2.2*, *SaNRT2.1*, and *SaNRT2.5* genes were amplified from the previously obtained constructs pSPYCE-35S-SaNAR2.2, pSPYNE-35S-SaNRT2.1, and pSPYNE-35S-SaNRT2.5, respectively, using the CloneAmp HiFi PCR Premix kit and the primers listed in the Supplementary Table S1. The amplicons of the genes under investigation were ligated with the linear forms of vectors pMetYC-Dest or pNX35-Dest using the ClonExpress Ultra One Step kit (Cat. No. C115, Vazyme, Nanjing, China), resulting in three constructs: pMetYC-Dest-SaNAR2.2, pNX35-Dest-SaNRT2.1, and pNX35-Dest-SaNRT2.5. Construct pMetYC-Dest-SaNAR2.2 (bait vector) contained a sequence encoding the SaNAR2.2 protein fused at its C-terminus with CubPLV as bait, and constructs pNX35-Dest-SaNRT2.1 and pNX35-Dest-SaNRT2.5 (prey vectors) contained the SaNRT2.1/SaNRT2.5 proteins fused at their N-terminus with NubG as prey.

In the same manner, the bait construct pMetYC-Dest-Exg2 was obtained and used as a negative interaction control. This construct contained the sequence encoding the transmembrane domain Exg2 of *S. cerevisiae* exoglucanase II.

The pMetYC-Dest-SaNAR2.2 or pMetYC-Dest-Exg2 constructs were used to transform the haploid strain THY.AP4, while the other two constructs (pNX35-Dest-SaNRT2.1 and pNX35-Dest-SaNRT2.5) were used to transform the haploid strain THY.AP5 using the PEG4000/LiAc method [40]. Yeast transformants were plated on the agarized selective media. Transformants THY.AP4 carrying bait vectors were plated on CSM_-LM_ agarized medium; transformants THY.AP5 carrying prey vectors were plated on CSM_-TUM_ medium [40]. The resulting yeast transformants were mated with each other, and THY.AP4/THY.AP5 diploids were selected on agarized medium CSM_-LTUM_ (without leucine, tryptophan, uracil, and methionine). To detect the interaction between SaNAR2.2 and SaNRT2.1/SaNRT2.5 in vivo, an overnight culture of selected diploid strains in CSM_-LTUM_ liquid medium was centrifuged for 15 s at 6000× *g*, the yeast cells were washed with sterile distilled water, resuspended in sterile water to an OD_600_ of 1 or 0.1, and plated on agarized CSM_-LTUM_ medium or CSM_-LTUMAH_ medium (lacking leucine, tryptophan, uracil, methionine, adenine, and histidine). To the latter, methionine at various concentrations (0.5, 5, 50 and 500 μM) was added. Yeast growth was assessed after 2–3 days of incubation at 30 °C.

### 2.8. Yeast Hansenula Polymorpha Complementation Assay with pCCUR2-SaNAR2.2 and pCHLX-SaNRT2.1/pCHLX-SaNRT2.5 Constructs

In these experiments, methylotrophic yeast *Hansenula polymorpha* double-auxotrophic strain DL-1 (*leu2 ura3* genotype) (wild-type strain, WT strain) and the mutant *H. polymorpha* strain *Δynt1* (Δ*ynt1: BleoR/ZeoR, leu2, ura3*) with a deleted gene *YNT1* encoding the only high-affinity nitrate transporter in *H. polymorpha* were used. The mutant strain *Δynt1* was obtained by us earlier [49].

To study the function of SaNAR2.2 protein and the SaNRT2.1/SaNRT2.5 proteins, the mutant *H. polymorpha* strain *Δynt1* was co-transformed with pairs of integrative vector constructs: pCHLX-SaNRT2.1/pCCUR2, or pCHLX-SaNRT2.5/pCCUR2, or pCHLX-SaNRT2.1/pCCUR2-SaNAR2.2, or pCHLX-SaNRT2.5/pCCUR2-SaNAR2.2. Yeast integrative vectors pCCUR2 and pCHLX [53] carrying the *URA3* and *LEU2* genes, respectively, ensure the growth of the yeast strains in the selective media without additional nitrogen sources, namely leucine and uracil, when performing complementation tests. Plasmid pCCUR2 was constructed by deletion of the KpnI-XbaI fragment in the pCCUR1 plasmid [54].

Cells of the *H. polymorpha* WT strain and the mutant *Δynt1* strain were routinely grown in a rich YPD medium (1% yeast extract, 2% peptone, 2% glucose). After transformation with vector constructs, yeast cells were grown in a minimal synthetic SD medium (0.17% yeast nitrogen base without amino acids and ammonium sulfate, 2% glucose) with the addition of 0.5% (NH_4_)_2_SO_4_ as a nitrogen source. All agarized media contained 2% agar. Yeast transformants containing genomic inserts were verified using PCR.

The constructs pCHLX-SaNRT2.1 and pCHLX-SaNRT2.5 were obtained by us earlier [42]. In these constructs, coding sequences *SaNRT2.1* and *SaNRT2.5* were under the control of the inducible nitrate reductase (YNR1) promoter *pYNR1* and terminator *tYNR1* of *H. polymorpha*. Wild-type strain DL-1 and the mutant strain *Δynt1* co-transformed with vector pair pCHLX/pCCUR2 were taken as controls. Vector constructs were linearized at the PspEI site (for pCHLX and pCHLX-SaNRT2.5), EgeI site (for pCHLX-SaNRT2.1), or PspCI site (for pCCUP2 and pCCUR2-SaNAR2.2) and *H. polymorpha* cells were transformed with the linear plasmid forms. Yeast cells were transformed by electroporation [55] using an Eppendorf device (Eppendorf, Framingham, MA, USA). Yeast transformants were selected on minimal selective synthetic SD medium, in the absence of leucine and uracil.

Selected yeast transformants were grown in liquid SD medium containing 0.5% ammonium sulfate overnight at 37 °C. Then, 1 mL of the yeast cultures was centrifuged for 5 min at 2500× *g*; the cell precipitates were washed with sterile water, resuspended in 1 mL water; and 2 μL of each of the obtained suspensions was placed on agarized SD medium containing different KNO_3_ concentrations (0.2, 0.5, 1.0, 2.5, and 5.0 mM). The plates were incubated at 37 °C for 2–3 days until colonies appeared.

### 2.9. Vector Constructs pCCUR2-SaNAR2.2-mCherry and pCHLX-SaNRT2.5-GFP

For the expression of SaNAR2.2 fused at the C-terminus with red fluorescent protein mCherry and SaNRT2.5 fused at the C-terminus with green fluorescent protein GFP in *H. polymorpha* cells, the pCCUR2-SaNAR2.2-mCherry and pCHLX-SaNRT2.5-GFP constructs were obtained where coding sequences *SaNAR2.2::mCherry* and *SaNRT2.5::GFP* were under the control of the inducible nitrate reductase promoter *pYNR1* and terminator *tYNR1* of *H. polymorpha*. GFP coding sequence was amplified from plasmid pTR-UF12 [56] with GFP_Gib_F and tYNR1_GFP_R primers. mCherry coding sequence was amplified from pcDNA3.1/hChR2(H134R)-mCherry plasmid with mCherry_Gib_F and tYNR1_mCherry_R primers. pCHLX-SaNRT2.5 and pCCUR2-SaNAR2.2 constructs were linearized by inverse PCR with corresponding primers. Ligation of fluorescent protein coding sequences with linearized constructs was carried out using the Gibson assembly kit (NEB, Ipswich, MA, USA). Final coding sequences of interest in pCHLX and pCCUR2 vectors were verified by sequencing.

*H. polymorpha* cells were co-transformed with the linear forms of the constructs obtained as described in Section 2.8. The images of the transformed yeast cells were obtained with a laser scanning confocal microscope LSM-710-NLO (Carl Zeiss, Jena, Germany). The fluorescence images were obtained using excitation/emission wavelengths of 488/512 nm for GFP and 587/610 nm for mCherry.

### 2.10. Bioinformatics Analysis

The virtual translation of the nucleotide sequence *SaNAR2.2* into an amino acid sequence was carried out using the online service on the ExPASy portal (http://web.expasy.org/translate/ (accessed on 2 November 2024)). The molecular weight of the SaNAR2.2 protein was also determined using the service on this portal (http://web.expasy.org/protparam/ (accessed on 2 November 2024)). The cellular localization of the protein was predicted using the online service WoLF PSORT II on the GenScript portal (https://www.genscript.com/wolf-psort.html (accessed on 2 November 2024)). The affiliation of the SaNAR2.2 protein with the NAR2/NRT3 family and the prediction of its functions were carried out using the InterPro v.102.0 resource (http://www.ebi.ac.uk/interpro/ (accessed on 2 November 2024)) as well as the Protein BLAST algorithm on the NCBI portal (http://www.ncbi.nlm.nih.gov/ (accessed on 2 November 2024)). The transmembrane topology of the protein was predicted using online DeepTMHMM software v.1.0.24 (https://dtu.biolib.com/DeepTMHMM (accessed on 2 November 2024)). The 3D-structure models and the model of interaction between SaNAR2.2 and SaNRT2.5 proteins were created using AlphaFold3 software [57] at https://alphafoldserver.com (accessed on 2 November 2024).

Multiple alignment of amino acid sequences of NAR2 family proteins was performed online using the MAFFT v.7 software (https://www.ebi.ac.uk/Tools/msa/mafft/ (accessed on 2 November 2024)) and visualized using Jalview v. 2.11.2.7 software (https://www.jalview.org/ (accessed on 2 November 2024)). Phylogenetic analysis of NAR2 family proteins was performed using the Molecular Evolutionary Genetic Analysis software (MEGA v.11; https://www.megasoftware.net/ (accessed on 2 November 2024)), employing the maximum likelihood method based on the Jones–Taylor–Thornton model [58] (1000 bootstrap replicates were performed). Amino acid sequences of NAR2 family proteins for comparative analysis were extracted from the NCBI portal (https://www.ncbi.nlm.nih.gov/protein/ (accessed on 2 November 2024)).

## 3. Results

### 3.1. Cloning of the Full-Length Coding Sequence of SaNAR2.2 and Bioinformatic Analysis of the SaNAR2.2 Protein

In early stages of our search for sequences encoding nitrate-transporting proteins of the halophyte *S. altissima*, a partial coding sequence of the *NAR2* family gene from *S. altissima* was identified [47]. In the present study, gene-specific primers based on the previously identified partial sequence were designed, and the 3′- and 5′-end fragments of the full-length coding sequence (CDS) of the *NAR2* family gene from *S. altissima* were amplified using the Step-Out RACE method (Figure S5A,B); then the full-length targeted cDNA of 591 bp was amplified (Figure S5C). For amplification of the target sequence, first-strand cDNA synthesized from total RNA extracted from *S. altissima* roots was used, as it is known that *NAR2* genes are predominantly expressed in plant roots [59,60]. The *SaNAR2* full-length CDS of 591 bp was cloned into the yeast integrative plasmid pCCUR2 for further expression in the cells of yeast *H. polymorpha*.

**Figure 1:**
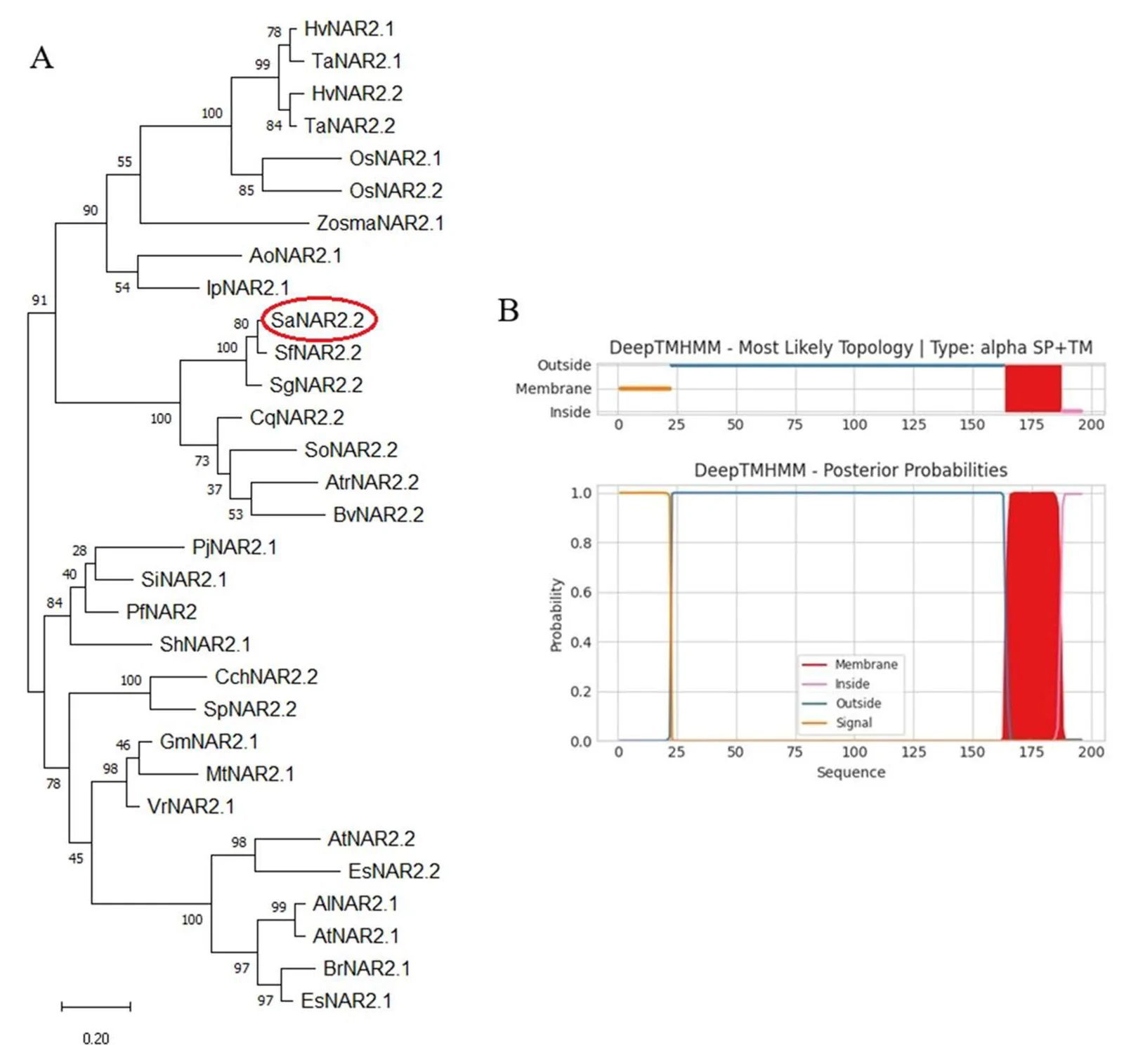
(**A**) An unrooted phylogenetic tree of plant NAR2 proteins including SaNAR2.2 from *S. altissima*. The phylogenetic tree was built in MEGA v.11 using the maximum likelihood method based on the Jones–Taylor–Thornton model. The number of bootstrap replicates was 1000; the values of bootstrap support are indicated near the nodes. The NAR2 protein sequences were extracted from the NCBI portal (https://www.ncbi.nlm.nih.gov/protein/, accessed on 15 October 2024). Names of plant species and protein IDs are given in Supplementary Table S2. (**B**) Two-dimensional topology of the SaNAR2.2 protein predicted using DeepTMHMM software (v.1.0.24). SaNAR2.2 has one transmembrane segment in the C-terminal region (164–187 aa). The large N-terminal part of SaNAR2.2 is located in the extracellular space. A signal sequence (1–22 a.a.) is also present at the N-end.

The cloned sequence (deposited in GenBank, ID: OR828749.1) contained an open reading frame for a protein of 196 amino acids in size, with a calculated molecular mass of 21.7 kDa (GenBank ID: WPH61291.1). The affiliation of this protein with the NAR2/NRT3 family was confirmed using the online resource InterPro. On the phylogenetic tree of NAR2 family proteins, the protein from *S. altissima* is located in a clade with proteins from the NAR2.2 subfamily found in other species of the Amaranthaceae (Figure 1A). Therefore, on the basis of phylogenetic relationships, we classified the protein into this subfamily and named it SaNAR2.2.

According to the bioinformatic predictions of cellular localization and protein topology, SaNAR2.2 is likely a resident of the plasma membrane; it has one transmembrane segment at the C-terminal region (164–187 aa), and a small C-end (188–196 aa) located in the cytoplasm. The large N-terminal part of the protein SaNAR2.2 (1–163 aa) is located in the extracellular space; a signal sequence (1–22 aa) was also identified at the N-terminus (Figure 1B).

**Figure 2:**
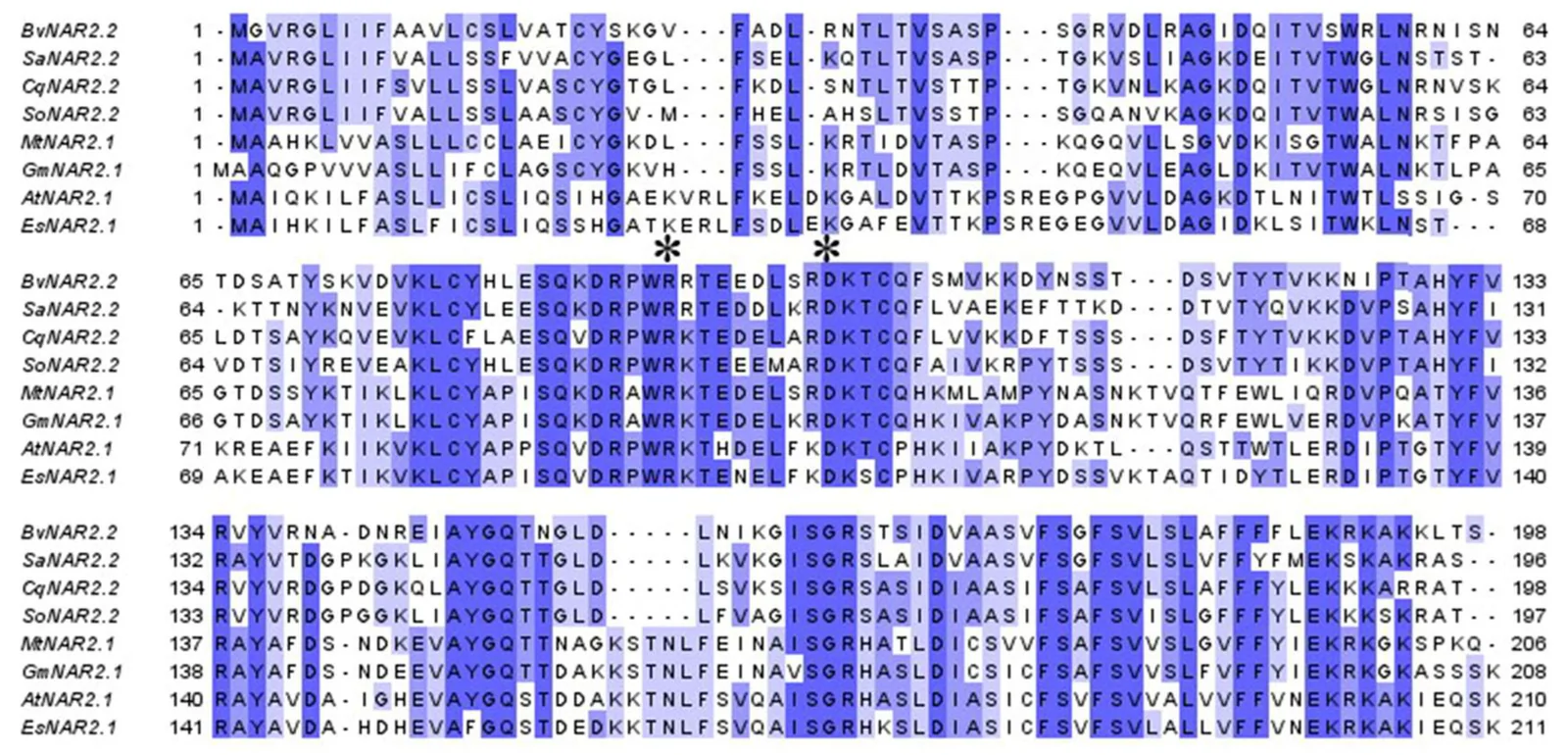
Multiple sequence alignment performed in Clustal Omega software (https://www.ebi.ac.uk/jdispatcher/msa/clustalo, accessed on 22 October 2024) for NAR2 proteins from *Arabidopsis thaliana* (AtNAR2.1: NP_199831.1), *Beta vulgaris* (BvNAR2.2: XP_010667500.1), *Chenopodium quinoa* (CqNAR2.2: XP_021720161.1), *Eutrema salsugineum* (EsNAR2.1: XP_006402168.1), *Glycine max* (GmNAR2.1: XP_003549822.1), *Medicago truncatula* (MtNAR2.1: XP_013457733.1), *Spinacia oleracea* (SoNAR2.2: XP_021863523.1), and *Suaeda altissima* (SaNAR2.2: WPH61291.1). Protein GenBank IDs are indicated in the parentheses. The key amino acid residues, which are presumably crucial for interaction with the transporters of the NRT2 family, are marked by asterisks. The intensity of the staining of amino acid residues corresponds to the degree of their identity (percentage identity).

The alignment of the amino acid sequence of the SaNAR2.2 protein with the sequences of some other NAR2 proteins showed that proteins of this family from various plant sources possess conserved domains in the central part and in the C-terminal region (Figure 2). The findings suggest that the amino acid sequences in these regions are likely involved in interactions with the partner proteins of the NRT2 family.

It has been shown that in their central region, NAR2 proteins have important conserved residues of arginine R and aspartate D, which are crucial for interaction with NRT2 family transporters [61,62]. The position of these residues in different NAR2 proteins varies slightly. For example, these are R96 and D105 for the AtNAR2.1 from *A. thaliana* [61] and R100 and D109 for the OsNAR2.1 from rice [62]. In the central region of the SaNAR2.2, these conserved arginine and aspartate residues apparently correspond to the amino acid residues R88 in the conserved motif WR(K,R) and D97 in the motif RDKTCQ (Figure 2). The modeling of the three-dimensional structures of SaNAR2.2 (Figure S6A), SaNRT2.5 (the high-affinity nitrate transporter of the NRT2 family from *S. altissima*, presumably a partner protein for SaNAR2.2) (Figure S6B), and the heterodimer of these proteins (Figure S6C) also predicted that the central region of SaNAR2.2 is involved in interaction with the protein SaNRT2.5. Moreover, the predicted 3D model of the heterodimer demonstrated that the transmembrane C-terminal region of the SaNAR2.2 protein and a small C-end domain located in the cytoplasm (164–196 aa), which form an α-helix, may also be involved in this interaction (Figure S6C).

### 3.2. Detection of the Interaction Between SaNAR2.2 and SaNRT2.1/SaNRT2.5 Proteins

The question of whether the protein SaNAR2.2 associates with the potential high-affinity nitrate transporters SaNRT2.1/SaNRT2.5 from *S. altissima* was clarified using the BiFC method in *N. benthamiana* leaves and yeast mating-based split-ubiquitin assay (mbSUS).

**Figure 3:**
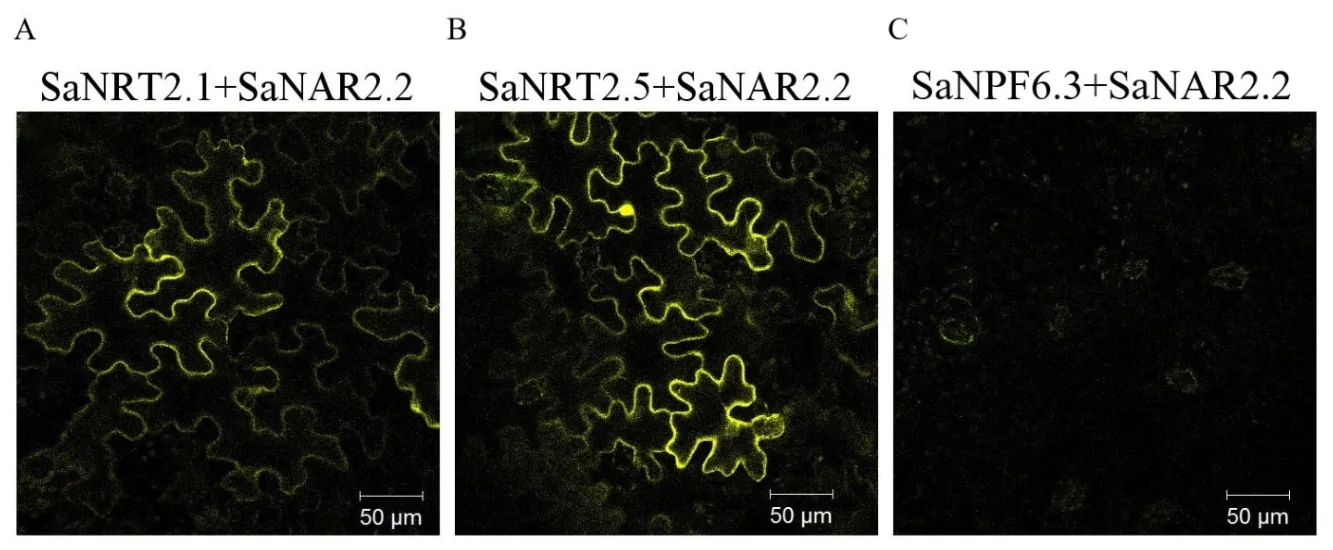
Detection of the SaNAR2.2/SaNRT2.1 (**A**) or SaNAR2.2/SaNRT2.5 (**B**) interactions in leaves of *N. benthamiana* plants using the BiFC method. Transient co-expression of SaNAR2.2 fused with the C-terminal half of the fluorescent protein mVenus and SaNRT2.1 or SaNRT2.5, each fused with the N-terminal half of mVenus, in *N. benthamiana* leaves resulted in the recovery of mVenus fluorescence (**A**,**B**), observed at the periphery of transformed tobacco leaf cells. The images of the fluorescence signal were obtained with a laser scanning confocal microscope LSM-710-NLO (Carl Zeiss, Jena, Germany) at λ_ex_ 515 nm and λ_em_ 527 nm. In these experiments, SaNPF6.3, a low-affinity nitrate transporter of the NRT1 family from *S. altissima*, was co-expressed with SaNAR2.2 in the leaves of *N. benthamiana* as a negative control (**C**). Each confocal image is a representative picture of eight independent assays.

The BiFC assay is based on the recovery of fluorescence when two non-fluorescent N-and C-terminal halves of a fluorescent protein are brought together [39]. The genetic constructs for analyzing the interaction of SaNAR2.2 with SaNRT2.1 and SaNRT2.5 were obtained based on plasmid vectors that encode, respectively, the C-terminal and N-terminal halves of the yellow fluorescent protein mVenus. Using the vector constructs, transient expression of the specified *S. altissima* genes was achieved in *N. benthamiana* leaves. Co-expression of SaNAR2.2, fused with the C-terminal fragment of mVenus, and SaNRT2.1 or SaNRT2.5, each fused with the N-terminal fragment of mVenus, resulted in the recovery of mVenus fluorescence (Figure 3A,B). The latter demonstrated that two non-fluorescent N- and C-terminal halves of the fluorescent protein were brought together through the interaction of SaNAR2.2 with SaNRT2.1 or SaNRT2.5.

The fluorescence of mVenus could be observed at the periphery of transformed tobacco cells, suggesting localization of the interacting proteins SaNRT2/SaNAR2.2 at the plasma membrane. In these experiments, *SaNPF6.3/SaNRT1.1* (GenBank ID: OQ330855.1), a low-affinity nitrate transporter gene of the *NPF/NRT1* family from *S. altissima* [44], was co-expressed with *SaNAR2.2* in the leaves of *N. benthamiana* as a negative control. Since NAR2 family proteins interact with NRT2 proteins but not with NPF/NRT1 proteins, no mVenus fluorescence was observed in tobacco leaves co-transformed with the construct pSPYCE-35S-SaNAR2.2 and the construct pSPYNE-35S-SaNPF6.3 carrying the *SaNPF6.3* sequence fused with the N-terminal fragment of mVenus (Figure 3C).

The potential interaction between the proteins SaNAR2.2 and SaNRT2.1/SaNRT2.5 was investigated using the yeast mating-based split-ubiquitin system (mbSUS) [40,63,64]. This mbSUS assay utilizes the ubiquitin-degradation pathway as a means to detect protein–protein interactions. The haploid yeast strain THY.AP4 was transformed with the construct pMetYC-Dest-SaNAR2.2, whereas the strain THY.AP5 was transformed with the constructs pNX35-Dest-SaNRT2.1 and pNX35-Dest-SaNRT2.5. After transformation, the yeast strain THY.AP4 contains the SaNAR2.2-CubPLV fusion protein as a bait, while strain THY.AP5 contains the NubG-SaNRT2.1 or NubG-SaNRT2.5 fusion protein as a prey. After mating of the yeast strains, in the diploid strain THY.AP4/THY.AP5, a functional ubiquitin will be reassembled if proteins SaNAR2.2 and SaNRT2.1/SaNRT2.5 interact with each other; this leads to cleavage of the recombinant transcription factor PLV. The released PLV switches on reporter genes *ADE2* and *HIS3*, which allows yeast growth on appropriate selective medium without adenine and histidine.

**Figure 4:**
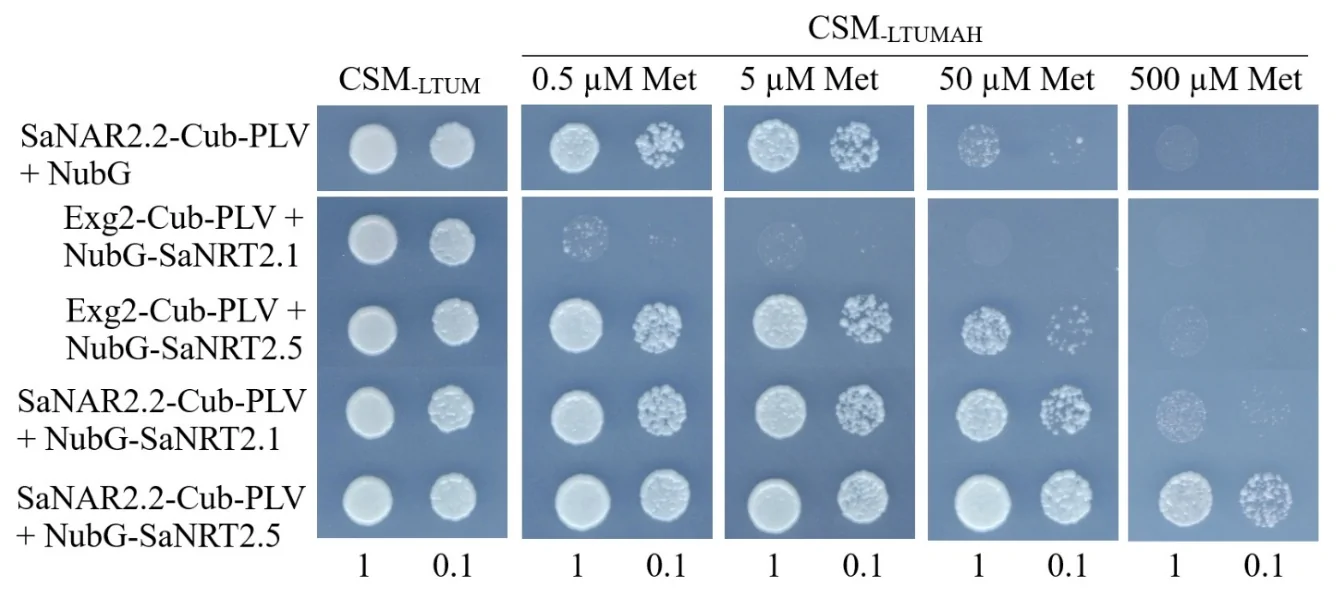
Diploid yeast strains THY.AP4/THY.AP5 expressing SaNAR2.2-CubPLV as a bait and NubG-SaNRT2.1 or NubG-SaNRT2.5 fusion proteins as a prey were spotted onto different agarized media as indicated. The medium CSM-LTUM was used to verify the presence of both bait and prey vectors. The addition of increasing concentrations of methionine (Met) to CSM-LTUMAH medium was used to verify the specificity of SaNAR2.2 interactions with the proteins. The negative controls used were “SaNAR2.2-Cub-PLV + NubG” (diploid yeast strain transformed by the construct encoding the SaNAR2.2-CubPLV as bait and the empty primary vector pNX35-Dest with NubG sequence for prey) and “Exg2-Cub-PLV + NubG-SaNRT2.1” and “Exg2-Cub-PLV + NubG-SaNRT2.5” (diploid yeast strains transformed by the construct pMetYC-Dest-Exg2 as bait vector and the constructs pNX35-Dest-SaNRT2.1 or pNX35-Dest-SaNRT2.5 as prey vectors). The results of a representative experiment with three biological replicates are presented.

In our experiments, diploid yeast strains that expressed SaNAR2.2-CubPLV along with NubG-SaNRT2.1 or NubG-SaNRT2.5 proteins demonstrated the ability to grow on a selective medium devoid of adenine and histidine (Figure 4). This growth indicated an interaction between the SaNAR2.2 protein and the SaNRT2.1/SaNRT2.5 proteins. For the negative control, we utilized a vector construct, pMetYC-Dest-Exg2, which encodes the transmembrane domain Exg2 of yeast exoglucanase II as a bait, to transform the THY.AP4 strain. Diploid yeast strains transformed by the constructs (pMetYC-Dest-Exg2 + pNX35-Dest-SaNRT2.1) or (pMetYC-Dest-Exg2 + pNX35-Dest-SaNRT2.5) showed different growth patterns on the selective medium CSM_-LTUMAH_. The strain expressing the proteins Exg2 and SaNRT2.1 was unable to grow on this medium, which clearly indicated the absence of interaction between these proteins, whereas the strain expressing the proteins Exg2 and SaNRT2.5 was able to grow on this selective medium (Figure 4). At the same time, an increase in methionine concentrations in the selective medium led to inhibition of the growth of the yeast strain expressing the proteins Exg2 and SaNRT2.5. In the experimental system used, methionine is the component that reduces the level of non-specific interactions, so we can assume that the growth of the diploid yeast strain in which Exg2 and SaNRT2.5 proteins are expressed is due to the non-specific interactions of these proteins. A diploid yeast strain containing SaNAR2.2 and SaNRT2.5 proteins grew most intensively on a selective CSM_-LTUMAH_ medium, and the growth of this strain was only slightly suppressed by high concentrations of methionine. The latter allows us to conclude that there is a strong and specific interaction between the SaNAR2.2 and SaNRT2.5 proteins. Interaction between the SaNAR2.2 and SaNRT2.1 proteins is likely less effective (Figure 4).

### 3.3. Expression of SaNAR2.2 in S. altissima Organs Under Various Ionic Conditions

Expression of the *SaNAR2.2* gene in the organs of *S. altissima* plants, grown under low (0.5 mM) and high (15 mM) nitrate concentrations in the nutrient medium in the absence of NaCl or in the presence of increasing salt concentrations (250 mM, 500 mM, 750 mM NaCl), was analyzed using the qRT-PCR method.

**Figure 5:**
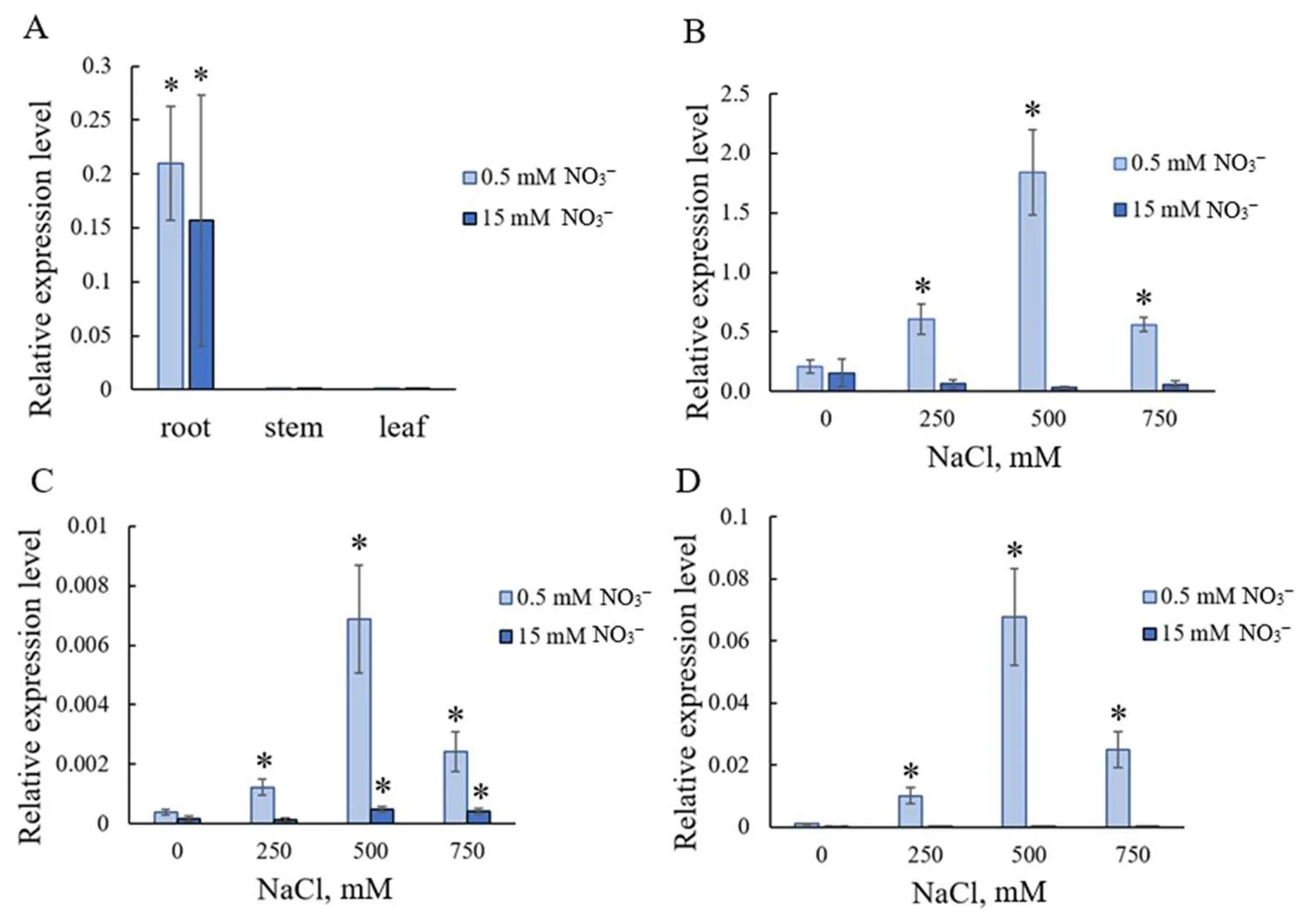
Relative abundance of *SaNAR2.2* transcripts (**A**,**B**), *SaNRT2.1* transcripts (**C**), and *SaNRT2.5* transcripts (**D**) in the roots of *S. altissima* plants grown in nutrient medium containing low (0.5 mM) or high (15 mM) NO_3_^−^ concentrations under increasing NaCl concentrations. Changes in expression are relative to that of the reference gene (Sae*EF1a* gene from *S. altissima*). A *p*-value < 0.05 was considered to be statistically significant. * *p* < 0.05. The means of three biological replicates and standard deviations are presented.

It was found that the *SaNAR2.2* gene was expressed almost exclusively in roots; in leaves and stems, the gene expression level was negligible (Figure 5A). In the absence of salt in the medium, the expression of *SaNAR2.2* was at comparable levels in the roots of plants grown at low (0.5 mM) or high (15 mM) NO_3_^−^ concentrations. With increasing salt concentration in the medium, the expression of *SaNAR2.2* decreased when the nutrient medium contained high concentrations of nitrate, and conversely, the gene expression sharply increased at low nitrate concentrations in the nutrient medium, reaching maximum values at 500 mM NaCl. At this salt concentration, the level of gene expression increased almost tenfold (Figure 5B). Further increase in NaCl concentration in the nutrient solution to 750 mM led to a decrease in the expression of the *SaNAR2.2* gene.

Likewise, with low nitrate (0.5 mM) and increasing NaCl (to 500 mM), the expression of *SaNRT2.1* and *SaNRT2.5* (which encode the potential partners of the SaNAR2.2 protein, high-affinity nitrate transporters of the NRT2 family in *S. altissima,* and whose expression also occurs predominantly in *S. altissima* roots [37]) changed in a similar way (Figure 5C,D). However, the expression levels of these three genes, *SaNAR2.2*, *SaNRT2.1*, and *SaNRT2.5*, differed significantly. The expression of *SaNAR2.2* almost doubled that of the housekeeping gene *SaeEF1alfa*, whereas the expression levels of *SaNRT2.1* and *SaNRT2.5* were significantly lower.

### 3.4. Yeast Complementation Analysis of SaNAR2.2 Function

**Figure 6:**
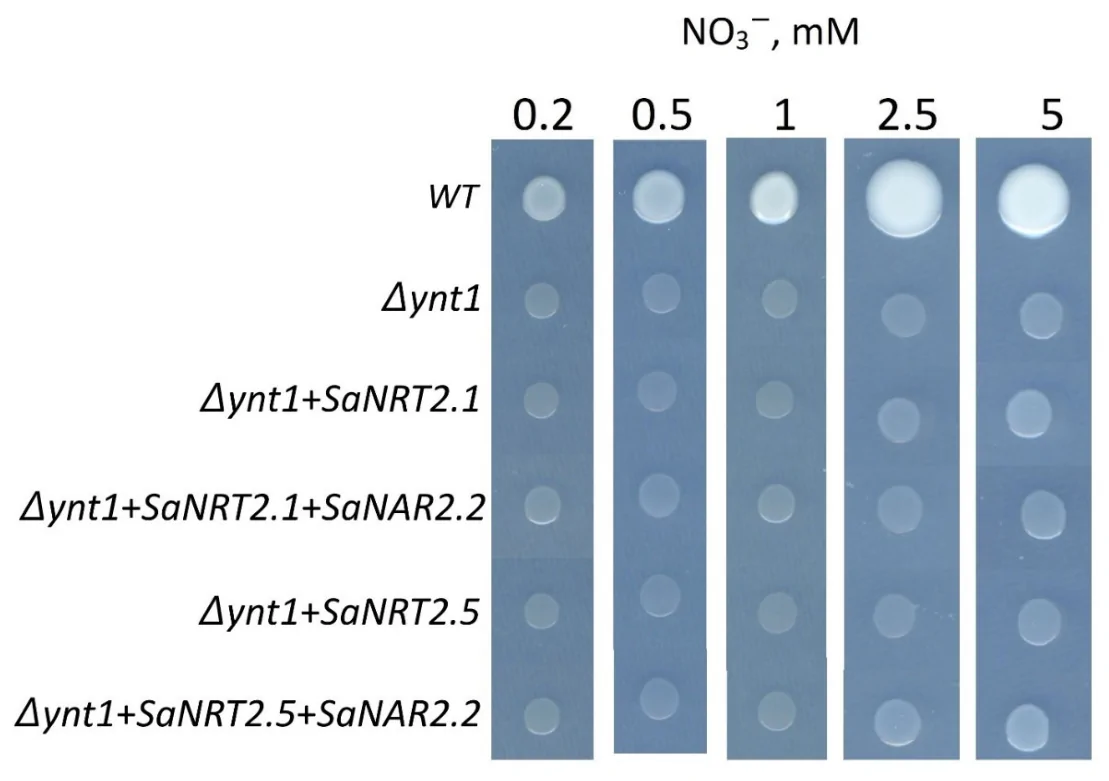
Complementation assay of the *H. polymorpha* mutant strain *Δynt1* co-transformed with pairs of integrative constructs: pCHLX-SaNRT2.1/pCCUR2, or pCHLX-SaNRT2.5/pCCUR2, or pCHLX-SaNRT2.1/pCCUR2-SaNAR2.2, or pCHLX-SaNRT2.5/pCCUR2-SaNAR2.2. Wild-type *H. polymorpha* strain DL-1 and the mutant strain *Δynt1* co-transformed with vector pair pCHLX/pCCUR2 were taken as controls. Yeast grew on minimal agarized SD medium supplied with indicated concentrations of NO_3_^−^. Approximately 10^5^ cells of each strain were plated on Petri dishes and incubated at 37 °C for 3 days. The results of a representative experiment with three biological replicates are presented.

To determine the possible functional role of SaNAR2.2 in complex with SaNRT2.1 or SaNRT2.5 proteins in NO_3_^−^ transport, the cells of the yeast *H. polymorpha* mutant strain *Δynt1*, which lacks the only gene for the nitrate transporter in this organism, were transformed with plasmids carrying *SaNRT2.1* or *SaNRT2.5* coding sequences, or co-transformed with the pair of plasmid constructs carrying *SaNRT2.1*/*SaNRT2.5* and *SaNAR2.2* coding sequences. All the yeast strains were plated on agarized nutrient media containing a range of nitrate concentrations (Figure 6). The growth of mutant strain *Δynt1* on the media containing nitrate as a nitrogen source was expectedly suppressed. Unfortunately, we did not see a clear picture of the expected recovery of growth of the mutant *Δynt1* transformed with vector constructs carrying *SaNRT2.1* or *SaNRT2.5* sequences or co-transformed with two plasmids carrying SaNAR2.2 and SaNRT2.1/SaNRT2.5 on these media. The growth of yeast transformants was significantly weaker than the growth of wild-type cells with an intact nitrate uptake mechanism.

**Figure 7:**
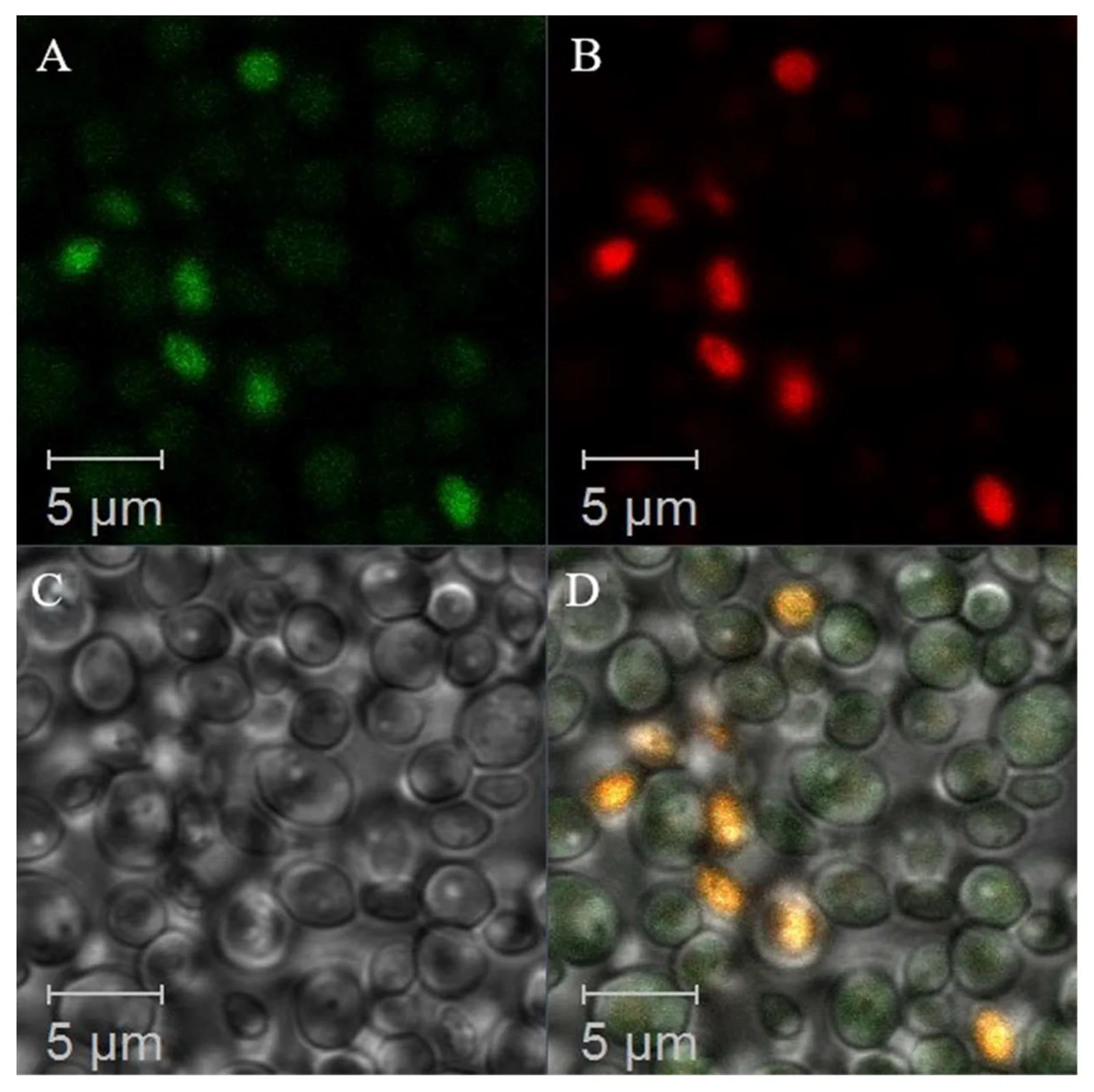
Images of *Δynt1* cells co-transformed with pCHLX-SaNRT2.5-GFP and pCCUR2-SaNAR2.2-mCherry constructs. (**A**) Green fluorescence of GFP. (**B**) Red fluorescence of mCherry. (**C**) Bright-field image. (**D**) Merge of GFP (**A**) and mCherry (**B**) fluorescence images (yellow color) and the bright-field image (**C**). The images were obtained with a laser scanning confocal microscope, LSM-710-NLO (Carl Zeiss, Jena, Germany). The fluorescence images were obtained using excitation/emission wavelengths of 488/512 nm for GFP and 587/610 nm for mCherry, respectively. The image is a representative picture of ten independent assays.

The apparent lack of functional complementation of the yeast mutant strain *Δynt1* by *S. altissima* proteins may have been due to an inadequate level of the heterologous protein in yeast cells (apart from the other possible explanations). To determine the presence of recombinant *S. altissima* proteins in the transformed yeast cells, the mutant *Δynt1* was co-transformed with vector constructs pCCUR2-SaNAR2.2-mCherry and pCHLX-SaNRT2.1-GFP/pCHLX-SaNRT2.5-GFP. The plasmids carried coding sequences, accordingly, for SaNAR2.2 fused at the C-terminus with red fluorescent protein mCherry, and for SaNRT2.1/SaNRT2.5 fused at the C-terminus with green fluorescent protein GFP. Co-expression of *S. altissima* transgenes was observed in transformed yeast cells. This was confirmed through qRT-PCR experiments (Figure S7) and by examining the transformed yeast cells with a laser scanning microscope (Figure 7). The proteins of interest were synthesized in only a small fraction of the transformed cells. Additionally, these proteins remained mainly confined to the internal space of the yeast cells without a distinct signal in the plasma membrane (Figure 7). The Discussion Section addresses potential causes for this outcome.

## 4. Discussion

In our work, the coding sequence of the *NAR2* family gene from the euhalophyte *S. altissima* was cloned. The search for the gene of interest in this plant was complicated by the fact that there is no sequenced genome of this species. Therefore, we used an in silico search in the de novo assembled transcriptomes of two related halophyte species, *S. fruticosa* and *S. glauca*, where we had previously identified the transcripts of *NAR2* family genes [47]. Transcripts of a single *NAR2* gene were found in both *S. fruticosa* and *S. glauca* transcriptomes. The full-length coding sequence of *SaNAR2.2* cloned in this work was obtained based on the homology of *NAR2* transcripts from *S. fruticosa* and *S. glauca*. Using primers derived from *NAR2* transcripts discovered in *S. fruticosa* and *S. glauca* transcriptomes, we found a single *NAR2* sequence for *S. altissima*. A single *NAR2* gene has also been found in a number of other plants: chrysanthemum [15], maize [17], seagrass *Zostera marina* [18], wheat [19], and spinach [20]. It should be noted that although several *NAR2* family genes have been identified in plant genomes, a single NAR2 protein is known to be involved in nitrate uptake via interactions with NRT2 proteins, as shown for AtNAR2.1 [10,21], OsNAR2.1 [14], and HvNAR2.3 [60]. Phylogenetic analysis demonstrated that the NAR2 protein from *S. altissima* belongs to the same clade as NAR2.2 proteins from other members of the Amaranthaceae (Figure 1A), and therefore, based on clustering with proteins currently annotated as NAR2.2 in the family, it was classified in this subfamily. However, it should be emphasized that the functional diversification of the NAR2 family remains insufficiently understood. This is largely due to the limited number of plant species in which NAR2 proteins have been functionally characterized. Therefore, the designation of SaNAR2.2 should currently be considered a phylogenetic annotation rather than a definitive functional classification, as no clear evolutionary or experimental criteria have yet been established to distinguish the roles of NAR2.1 and NAR2.2 proteins.

According to the secondary structure prediction, SaNAR2.2 has one transmembrane segment in the C-terminal region and a small C-terminal end located in the cytoplasm; the large N-terminal part of SaNAR2.2 is located in the extracellular space (Figure 1B).

To detect the interaction of SaNAR2.2 with putative partner proteins SaNRT2.1 and SaNRT2.5, previously identified high-affinity nitrate transporters in *S. altissima* [37], we used the BiFC (Figure 3) and mbSUS methods (Figure 4). The results obtained demonstrated that SaNAR2.2 associates with SaNRT2.1/SaNRT2.5, at least when heterologously expressed. The location of these interacting proteins in *S. altissima* cells still needs to be determined. However, it is known that orthologous proteins from other plants reside in the plasma membrane. Co-localization of NRT2 and NAR2 proteins in the plasma membrane of root cells has been demonstrated for barley [60] and Arabidopsis proteins [9,10,65].

The question of essential amino acid residues in NAR2 proteins that are involved in interaction with NRT2 proteins is of great interest. However, so far, a very limited number of studies have investigated this issue. For AtNAR2.1, it was demonstrated that the replacement of aspartate D105 in the central part of the protein markedly reduced nitrate uptake and accumulation in Arabidopsis plants [61]. Also, for OsNAR2.1, it was shown that mutations R100G and D109N disrupted the interaction of this protein with OsNRT2.3a [62], indicating that these amino acid residues are important not only for high-affinity nitrate transport activity but also for co-localization of OsNAR2.1 with OsNRT2.3a at the plasma membrane. These arginine and aspartate residues, which are at a similar distance of nine amino acid residues from each other, appear to be conserved in the central parts of all NAR2 proteins; corresponding amino acid residues, R88 and D97, were also found in the halophyte protein SaNAR2.2 (Figure 2).

According to a protein topology prediction, only one C-terminal transmembrane segment is present in SaNAR2.2, while the central part of SaNAR2.2, with conserved amino acid residues that are important for the interaction with the partner protein, is located in the extracellular space (Figure 1B). Therefore, it is reasonable to assume that interaction of SaNAR2 and SaNRT2.1/SaNRT2.5 is partially realized in the extracellular space. This assumption is confirmed by an in silico model of interaction between SaNAR2.2 and SaNRT2.5, which was created using AlfaFold3 software (Figure S6). In addition, the 3D model of the SaNAR2.2/SaNRT2.5 heterodimer suggests that the transmembrane C-terminal region of SaNAR2.2 may also be involved in this interaction (Figure S6).

Although transport-related functionality of SaNAR2.2 remains unconfirmed, we suggest the involvement of protein in the plant’s response to stressful conditions of low nitrate and high sodium chloride concentrations in the external medium. Apparently, the role of SaNAR2.2 as a participant in a high-affinity nitrate uptake system was particularly significant under saline conditions. *SaNAR2.2* was expressed almost exclusively in *S. altissima* roots (Figure 5A), similarly to *NAR2* genes from other plant species [11,12,17,21]. In the absence of salt in the nutrient solution, the expression levels of *SaNAR2.2* at low (0.5 mM) and high (15 mM) nitrate concentrations were similar (Figure 5A). However, with an increase in external NaCl concentration to 500 mM, the expression of SaNAR2.2 decreased at high nitrate concentration in the medium but significantly increased at low nitrate concentration (Figure 5B). Under low nitrate, the expression of *SaNRT2.1* and *SaNRT2.5*, which encode high-affinity nitrate transporters of the NRT2 family in *S. altissima* and are also observed mainly in *S. altissima* roots [37], changed in a similar way depending on changes in external NaCl concentrations (Figure 5C,D). Similar expression patterns of the three genes, *SaNAR2.2, SaNRT2.1*, and *SaNRT2.5*, depending on the changes in nitrate and salt concentrations in the environment, suggest that there is a functional relationship between the products of these genes—as has been shown for orthologous proteins from other plant species [9,10,21]. We recently showed that at low external nitrate concentrations the growth of *S. altissima* plants was predictably reduced compared to plants grown at high concentrations of nitrate [37]. However, at low external nitrate concentrations, plant weight and nitrate accumulation in the aboveground organs of *S. altissima* were maximal at a high salt concentration of 500 mM NaCl. This indicates the effective functioning of a high-affinity nitrate uptake system under conditions of potential competition between nitrate and chloride for nitrate-transporting proteins.

It is worth noting that under low nitrate and high salinity, the expression of *SaNAR2.2* reached an extremely high level, which was almost double the expression of the housekeeping gene *SaeEF1alfa*, while the expression levels of *SaNRT2.1* and *SaNRT2.5* were significantly lower (Figure 5). The expression of *SaNRT2.5* was almost 20 times lower than the expression of *SaNAR2.2*, and the expression of *SaNRT2.1*, in turn, was 10 times lower than the expression of *SaNRT2.5*. Such differences in the expression levels of the *SaNRT2.1*/*SaNRT2.5* genes and the *SaNAR2.2* gene are probably explained by the fact that NAR2 proteins play multiple roles in plants [11]. It is assumed that in addition to their role as a component of high-affinity nitrate transport systems, NAR2 proteins are necessary for the targeting, localization and stabilization of NRT2 transporters in the plasma membrane [62,65]. In addition, in rice plants, it has been shown that both high- and low-affinity nitrate transport were greatly impaired by *OsNAR2.1* knockdown [14]. This suggests that NAR2 proteins are also involved in the low-affinity nitrate transport, but the mechanisms of such involvement have not yet been studied. NAR2 proteins are also involved in the regulation of root growth. Knockdown of *OsNAR2.2* significantly repressed the primary root elongation and severely reduced the number of lateral roots under low nitrate concentrations [66,67]. NAR2 proteins are involved in suppressing lateral root formation in response to a high sucrose-to-nitrogen ratio in the environment [68], i.e., NAR2 is likely involved in the signaling pathway that regulates lateral root architecture. Thus, we can assume that in *S. altissima* the role of SaNAR2.2 is diverse.

It can also be supposed that the significantly higher *SaNAR2.2* expression in comparison to the expression of the nitrate transporter genes *SaNRT2.1* and *SaNRT2.5* is, in addition to other factors, related to the stoichiometry of formation of the heterologous complex of SaNRT2s and SaNAR2.2 proteins in *S. altissima*. For *A. thaliana*, it has been shown that a 150 kDa plasma membrane oligomeric complex of AtNRT2.5 and AtNAR2.1 is the major contributor to high-affinity nitrate influx [69]. Also, AtNRT2.1 forms a 150 kDa complex with AtNAR2.1 [9]. The 150 kDa oligomer has been proposed to contain a tetramer consisting of two subunits each of AtNRT2 and AtNAR2.1 [9,22]. It can be argued that *S. altissima* has a different stoichiometry for the formation of the heterologous complex of proteins SaNAR2.2 and SaNRT2, requiring more than one molecule of SaNAR2.2 per molecule of SaNRT2. If so, this could be one of the reasons for incomplete functional complementation of the yeast mutant strain *Δynt1* by SaNRT2.1/SaNRT2.5 and SaNAR2.2 heterologous expression (Figure 6). In yeast, the expression level of *SaNAR2.2* could be insufficient for the formation of a functional nitrate transport complex. In our work, we used integrative yeast plasmids, the number of copies of which integrated into the yeast genome is generally random and low. It is also possible that, at a sufficient level of expression, only a small portion of the synthesized heterologous proteins reached the plasma membrane of yeast cells. Indeed, we observed that heterologous *S. altissima* proteins synthesized in the yeast cells remained localized in the internal space of the yeast cell and did not reach the plasma membrane (Figure 7). These observations also suggest that *S. altissima* proteins may require post-translational modifications to exhibit their nitrate-transporting activity in the native system, similar to phosphorylation of serine residues at the N-terminal region of the AtNRT2.1 proteins in *A. thaliana*, to enhance the stability of the AtNRT2.1-AtNAR2.1 protein complexes and increase their transport activity [70,71].

## Data Availability

All data included in this study are available upon request by contacting the corresponding authors. The seeds of *Suaeda altissima* are available from the authors upon request. The cloned *SaNAR2.2* coding sequence was deposited in GenBank (*Suaeda altissima* nitrate assimilation-related protein mRNA, ID: OR828749.1). Database sources for sequence data used in phylogenetic analyses are described in Supplementary Table S2.

## Supplementary Materials

The following supporting information can be downloaded at: https://www.mdpi.com/article/10.3390/membranes16080267/s1, Figure S1. Electrophoresis of the amplicon consisting of the full-length coding sequence *SaNAR2.2* merged with the promoter *pYNR1* and terminator *tYNR1* and electrophoresis of the linear form of the vector pCCUR2. Figure S2. Electrophoresis of the pCCUR2-pYNR1-SaNAR2.2-tYNR1 construct. Figure S3. Genetic map of the pCCUR2-pYNR1-SaNAR2.2-tYNR1 construct. Figure S4. Coding sequence of the gene *SaNAR2.2* from *S. altissima* cloned in the pCCUR2 vector. Figure S5. Analysis of 5′-end and 3′-end fragments of the *SaNAR2.2* coding sequence synthesized on the total cDNA template from *S. altissima* roots using Step-Out RACE technology. Figure S6. Three-dimensional structure modeling of SaNAR2.2 and SaNRT2.5 proteins, and SaNAR2.2/SaNRT2.5 heterodimer predicted by AlphaFold3 software. Figure S7. Relative abundance of *SaNAR2.2*, *SaNRT2.1*, and *SaNRT2.5* transcripts in the cells of *H. polymorpha* strain *Δynt1* co-transformed with (pCHLX-SaNRT2.1-GFP + pCCUR2-SaNAR2.2-mCherry) or (pCHLX-SaNRT2.5-GFP + pCCUR2-SaNAR2.2-mCherry) constructs. Table S1. List of the primers used in the research. Table S2. List of NAR2 proteins from the phylogenetic tree of Figure 1.
