# Supplementary material for: SaNAR2.2, A Novel Member of the NAR2 Family from the Euhalophyte Suaeda altissima: Molecular Cloning and Characterization
Source: Membranes (Basel). 2026 Aug 12;16(8):267. doi: 10.3390/membranes16080267 (PMC13515975; doi:10.3390/membranes16080267)
Supplement: Supplementary file 1 [file membranes-16-00267-s001.zip › membranes-4396406-supplementary.pdf]

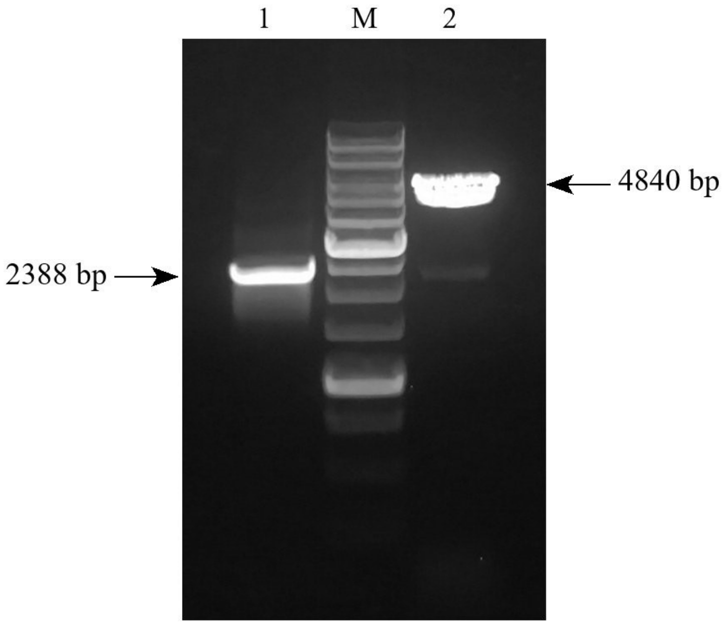

Figure S1. Electrophoresis of the amplicon consisting of the full-length coding sequence *SaNAR2.2* merged with the promoter *pYNR1* and terminator *tYNR1*, of a total length of 2388 bp, and the linear form of the vector pCCUR2 with a size of 4840 bp (cut at the KpnI restriction site). 1 – amplicon *pYNR1-SaNAR2.2-tYNR1*, 2 – plasmid pCCUR2, M – DNA marker kit, 250 – 10000 bp (cat. No. M12, SibEnzyme, Novosibirsk, Russia).

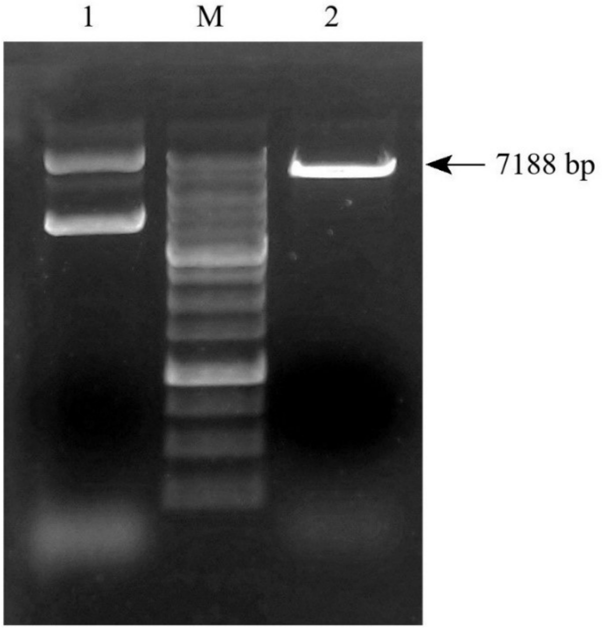

Figure S2. Electrophoresis of the pCCUR2-pYNR1-SaNAR2.2-tYNR1 construct. 1 – circular form of the construct; 2 – linear form of the construct after cutting at the unique restriction site PspCI; M – DNA marker kit, 250 – 10000 bp (cat. No. M12, SibEnzyme, Novosibirsk, Russia).

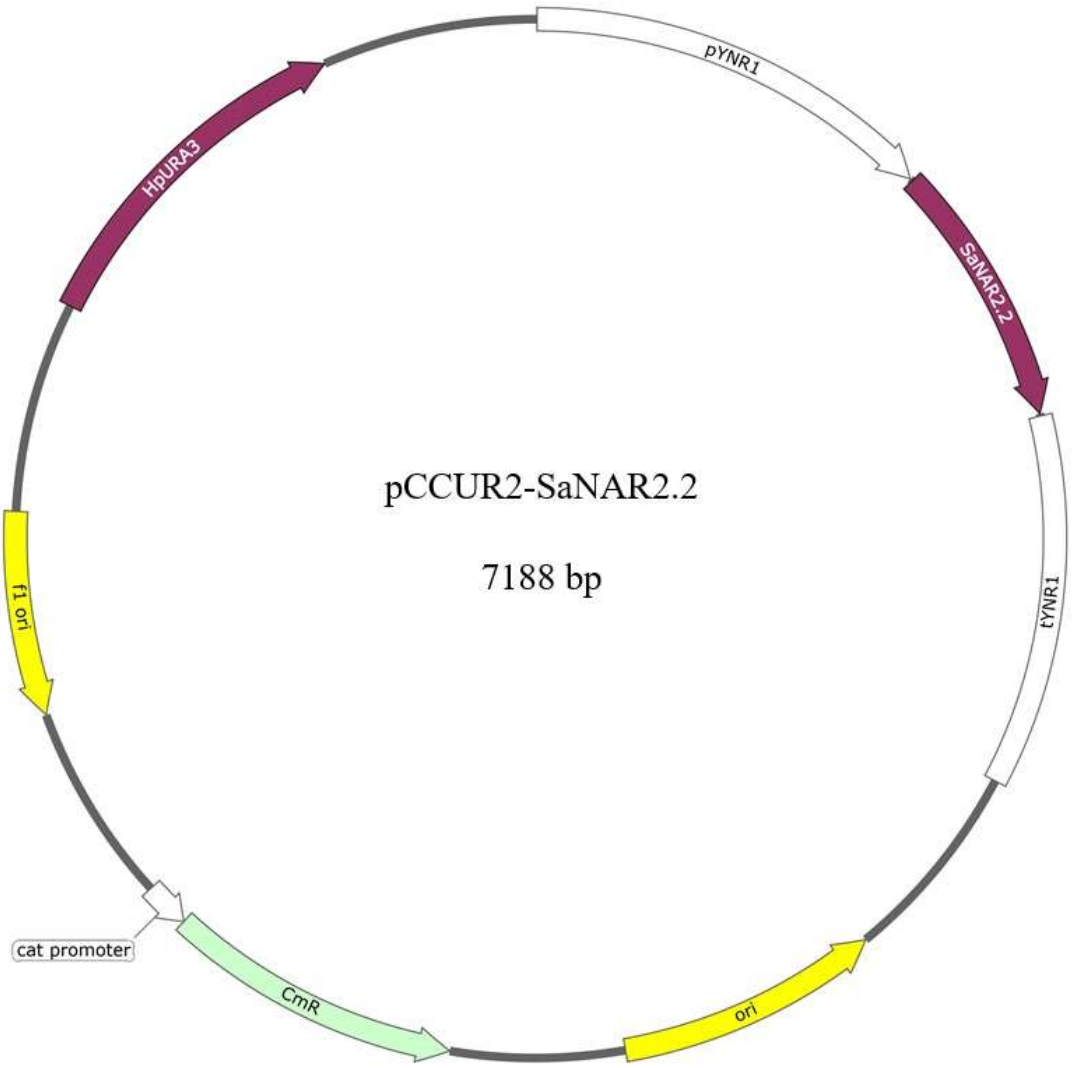

Figure S3. Genetic map of the pCCUR2-pYNR1-SaNAR2.2-tYNR1 construct. Ori – origins of replication, CmR – gene conferring resistance to the antibiotic chloramphenicol (chloramphenicol acetyltransferase), HpURA3 – auxotrophic marker (gene for uracil biosynthesis *URA3 H. polymorpha*), pYNR1 – promoter of the nitrate reductase gene *YNR1 H. polymorpha*, tYNR1 – terminator of the gene *YNR1 H. polymorpha*, SaNAR2.2 – coding region of the SaNAR2.2 gene *S. altissima*.

GATACGAATATAGTAGCTACTAAAAACCCTGCATGTAATTCCTCACGAACTTTGGATAG
TTGTAAGATCTATGGCGGTTCGTGGATTAATCATCTTTGTTGCACTTTTATCAAGCTTTG
TGGTAGCTTGTTATGGAGAAGGCTTGTTCTCCGAGCTTAAGCAAACTCTTACGGTCTC
TGCTTCTCCTACCGGAAAAGTGTCTCTAATAGCGGGGAAGGACGAAATCACCGTAAC
ATGGGGACTAAACAGTACATCAACGAAAACCACGAACTACAAGAACGTAGAGGTGA
AACTGTGCTACTTAGAAGAGAGCCAAAAGGACAGGCCATGGAGGAGAACCGAAGAC
GACTTGAAAAGAGACAAGACATGCCAATTTTTGGTAGCTGAAAAAGAATTTACCACA
AAAGATGACACAGTAACCTACCAAGTTAAAAAGGATGTTCCAAGTGCACATTACTTC
ATTAGGGCATACGTTAGCGATGGCCCTAAAGGGAAGCTTATCGCTTACGGCCAGACCA
CCGGTCTTGACCTTAAGGTCAAGGGCATCAGCGGCCGCAGCCTCGCCATCGACGTCG
CTGCTTCGGTTTTCTCAGGGTTTTCAGTTCTTTCTCTTGTGTTTTTCTACTTCATGGAG
AAGAGTAAGGCTAAGAGGGCTTCTTAAGCTAGCCCTAAGCGAAATCGAAATCAAAAC
TATCAAGAAACGTGGGTATGTTGTAGATATCAAGAACAGG

Figure S4. Coding sequence of the gene *SaNAR2.2* from *S. altissima* cloned in the pCCUR2 vector. The fragments of the yeast promoter *pYNR1* and terminator *tYNR1* (the full length sequences are merged with pCCUR2 vector) are highlighted. The coding sequence *SaNAR2.2* is deposited in GenBank, ID: OR828749.1.

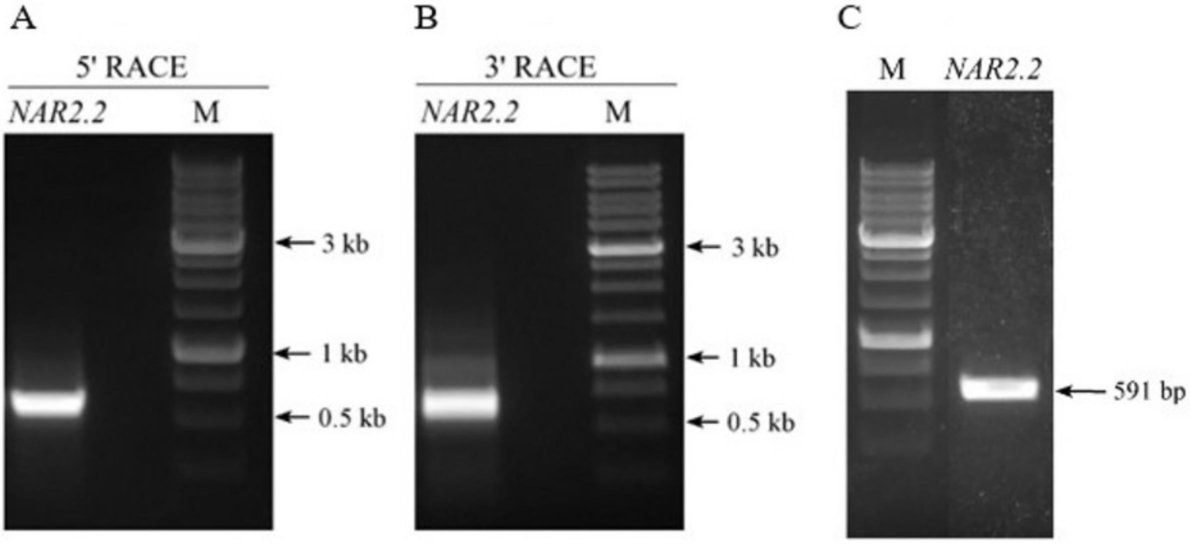

Figure S5. (A, B): Analysis of 5′-end and 3′-end fragments of *SaNAR2.2* coding sequence synthesized on the total cDNA template from *S. altissima* roots using Step-Out RACE technology. (C): Analysis of the full-length *SaNAR2.2* coding sequence used for further cloning. DNA fragments were separated by electrophoresis in 1% agarose gel. M — DNA molecular weight markers.

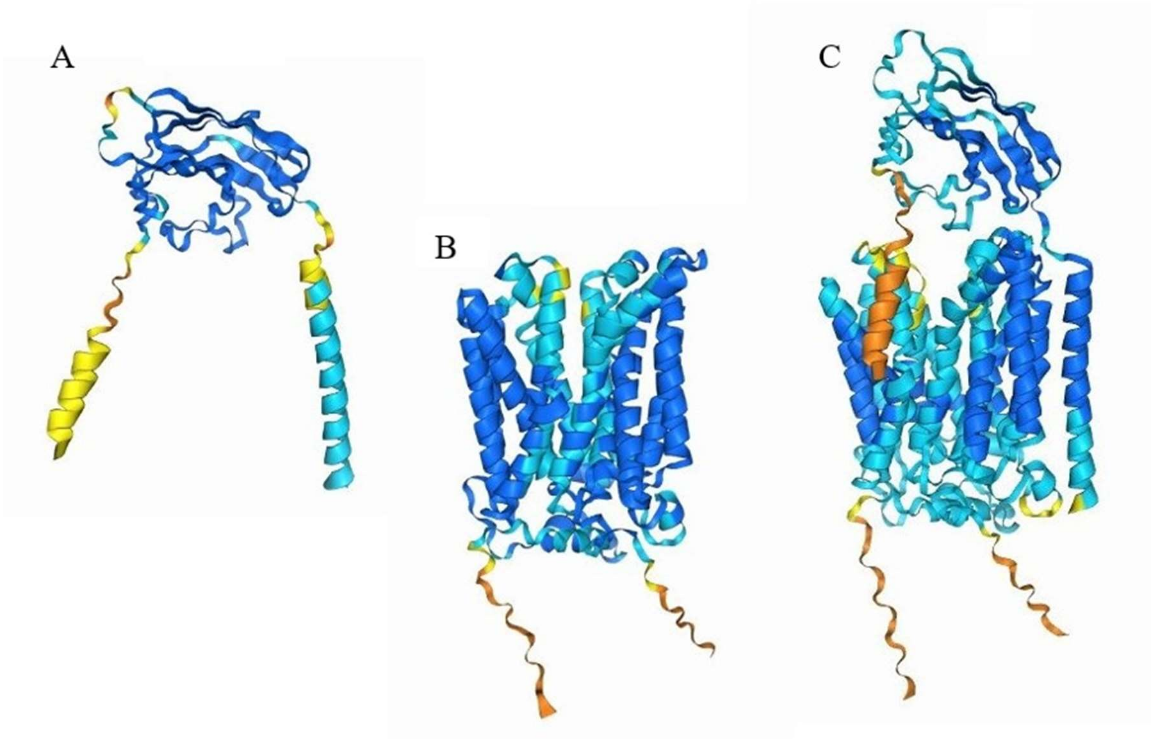

Figure S6. Three-dimensional structure modeling of SaNAR2.2 protein (A), SaNRT2.5 protein (B), and SaNAR2.2/SaNRT2.5 heterodimer (C) predicted by AlphaFold3 software (alphafoldserver.com). The model predicts that the central region of SaNAR2.2 and the transmembrane C-terminal region, which forms an α-helix, are involved in interaction with the protein SaNRT2.5.

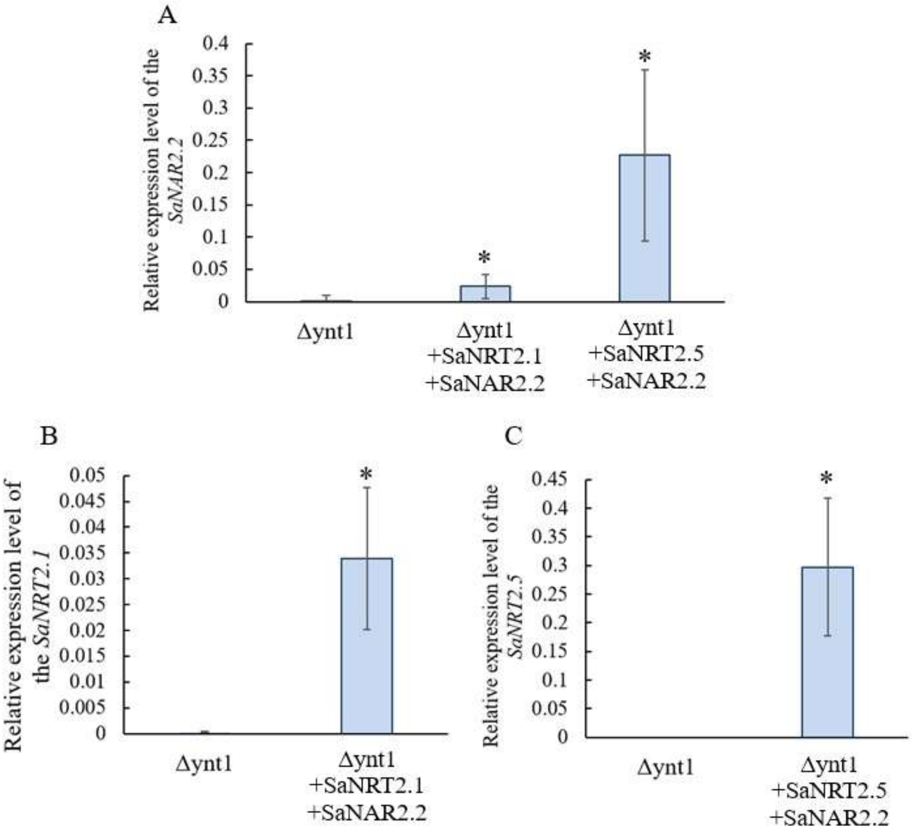

Figure S7. Relative abundance of *SaNAR2.2* transcripts (A), *SaNRT2.1* transcripts (B), and *SaNRT2.5* transcripts (C) in the cells of *H. polymorpha* mutant strain *Δynt1* co-transformed with (pCHLX-SaNRT2.1-GFP + pCCUR2-SaNAR2.2-mCherry) or (pCHLX-SaNRT2.5-GFP + pCCUR2-SaNAR2.2-mCherry) constructs. The changes in expression relative to the expression of the reference gene are presented; as a reference gene, the elongation factor gene *eEF1a* of *H. polymorpha* was used, the sequence of which was found in the transcriptome of *H. polymorpha* using the *eEF1a* gene of *Saccharomyces cerevisiae* as a query. A p-value < 0.05 was considered to be statistically significant. * $p < 0.05$. Standard deviations are given. The results of a representative experiment with three biological replicates are presented.

Table S1. List of the primers used in the research.

Primer name	Sequence
	5' RACE (I round)
SaNTR3.2_R	ACAATTAAGAAGCCCTTTTAGCCT
	3' RACE (I round)
SaNRT3.2_F	AAAAGTTAAGTAGTAATGGCGATTCGT
	5' RACE (II round)
SaNRT3.2_R1	CTTGGTAGGTTACTGTGTCATC
	3' RACE (II round)
SaNRT3.2_F1	CATGGAGGAGAACCGAAG
	SaNAR2.2 full-length CDS amplification
SaNRT3.2_F3	ATGGCGGTTCGTGGATTAATC
SaNRT3.2_R3	TTAAGAAGCCCTCTTAGCCTTACTC
	Linearization of vector pCHLX
pYNR1-SaNRT3.2_R	TCCACGAACCGCCATAGATCTTACAACTATCCAAAGTTCGTGAGG
tYNR1-SaNRT3.2_F	GCTAAGAGGGCTTCTTAAGCTAGCCCTAAGCGAAATCGAAATCAAAACT
	Amplification of the pYNR1-SaNAR2.2-tYNR1 fragment
pCCUR2-pYNR1-F	CTGCTTTTGTCTTGTGGTACTATGGGGCTCCATATATCGTATGAC
pCCUR2-tYNR1-R	CTAAAGGGAACAAAAGCTGGAATTCAGTATTTCAGAATCATGACCC
	Linearization of vector pMetYC-Dest
pMetYC-R	ATGGATTGTATGGATGGGGGTAATAG
pMetYC-F	GGCGCGCCGATTGGTGGTGGCGGA
	Amplification of SaNAR2.2, SaNRT2.1, SaNRT2.5 and SaNPF6.3 full-length CDS for mbSUS
pMetYC-SaNAR2.2-F	CCCATCCATACAATCCATATGGCGGTTCGTGGATTAATC
pMetYC-SaNAR2.2-R	ACCAATCGGCGCGCCAGAAGCCCTCTTAGCCTTACTC
pNX35-SaNRT2.1-F	CGGGCTATCCCTATGACGTCATGGCTGGTGAGCCG
pNX35-SaNRT2.1-R	TGCATAGTCCGGGACGTCCTATATTTGGTTAGAGTTGGGGCT
pNX35-SaNRT2.5-F	CGGGCTATCCCTATGACGTCATGGCATCAACAAATGAAGAATTTG
pNX35-SaNRT2.5-R	TGCATAGTCCGGGACGTCTTAGACACGATTAACTGTACTGCC
pNX35-SaNPF6.3-F	GGCTATCCCTATGACGTCATGGCTCATAATATTACTAGTATGGATATG
pNX35-SaNPF6.3-R	TGCATAGTCCGGGACGTCTTAGTGACATGCTGGCC
	Amplification of the sequence encoding the transmembrane domain of S. cerevisiae Exg2
pMetYC-Exg2-F	CCCATCCATACAATCCATATGCTTTCTAGTACCACCAC
pMetYC-Exg2-R	CCACCACCAATCGGCGCGCCCAGGGACGCGCACA

Table S2. List of NAR2 proteins*) from the phylogenetic tree of Fig. 2.

**Plant species**	**Protein name**	**GenBank ID**
Amaranthus tricolor	AtrNAR2.2	XP_057534669.1
Arabidopsis lyrata	AlNAR2.1	XP_020871335.1
Arabidopsis thaliana	AtNAR2.1	NP_199831.1
Arabidopsis thaliana	AtNAR2.2	NP_001190826.1
Asparagus officinalis	AoNAR2.1	XP_020246811.1
Beta vulgaris	BvNAR2.2	XP_010667500.1
Brassica rapa	BrNAR2.1	XP_009134048.2
Capsicum chinense	CchNAR2.2	PHU23681.1
Chenopodium quinoa	CqNAR2.2	XP_021720161.1
Eutrema salsugineum	EsNAR2.1	XP_006402168.1
Eutrema salsugineum	EsNAR2.2	XP_024005789.1
Glycine max	GmNAR2.1	XP_003549822.1
Hordeum vulgare	HvNAR2.1	AAP31850.1
Hordeum vulgare	HvNAR2.2	AAP31851.1
Iris pallida	IpNAR2.1	KAJ6792152.1
Medicago truncatula	MtNAR2.1	XP_013457733.1
Oryza sativa	OsNAR2.1	Q6ZI50.1
Oryza sativa	OsNAR2.2	Q7XK12.1
Perilla frutescens	PfNAR2	KAH6794745.1
Phtheirospermum japonicum	PjNAR2.1	GFQ07351.1
Salvia hispanica	ShNAR2.1	XP_047977271.1
Sesamum indicum	SiNAR2.1	XP_020548343.1
Solanum pennellii	SpNAR2.2	XP_015069077.1
Spinacia oleracea	SoNAR2.2	XP_021863523.1
Suaeda altissima	SaNAR2.2	WPH61291.1
Suaeda fruticosa	SfNAR2.2	Transcriptome assembl.
Suaeda glauca	SgNAR2.2	Transcriptome assembl.
Triticum aestivum	TaNAR2.2	AAV35211.1
Triticum aestivum	TaNAR2.1	AAV35210.1
Vigna radiata	VrNAR2.1	XP_014490580.1
Zostera marina	ZosmaNAR2.1	KMZ59910.1

*)Amino acid sequences of NAR2 family proteins for comparative analysis were extracted from the NCBI portal (https://www.ncbi.nlm.nih.gov/protein/).

```
>AtrNAR2.2
MAVRGLIIFAMIFSVMVTTCCGKVMFSHLANTLTVSTSPKGKVDLKAGVDSITITWGLNKNGSKVDTKSY
KNIEVKLCYSKESQKDRPWRKTEEELDRDKTCQFSVVKKEYNVTTDSFTYKVKKDVPTAHYFVRVYVRDG
PDGKQIAYGQTNGVDLNVTGISGRSSSIDIAASVFSTFSVVSLAFFFYLEKKKARKEEAK
```

```
>AlNAR2.1
MAIHKILFASLLICSLIQSSHGAEKVRLFKELDKGALDVTTQPSRQGDGVVLDAGKDTLNITWKLSAIGS
KREAEFKIIKVKLCYAPPSQLDRPWRKTHDELFKDKTCPHKIIAKPYDKTPQSTVWTVERDIPTGTYFVR
AYAVDAIGHEVAYGQSTDDAKTTNLFSVQAISGRHTSLDIASICFSVFSVVALVVFFVNEKRKAKIEQSK
```

>AtNAR2.1
MAIQKILFASLLICSLIQSIHGAEKVRLFKELDKGALDVTTKPSREGPGVVLDAGKDTLNITWTLSSIGS
KREAEFKIIKVKLCYAPPSQVDRPWRKTHDELFKDKTCPHKIIAKPYDKTLQSTTWTLERDIPTGTYFVR
AYAVDAIGHEVAYGQSTDDAKKTNLFSVQAISGRHASLDIASICFSVFSVVALVVFFVNEKRKAKIEQSK

>AtNAR2.2
MRHMCDTHLVVLPYQDHILIRIMAIHTLLFVSLLIFSLIESSSGGKKDRLFTDLQNSIEVTAKPVKDSGV
LEAGKDMVTITWKLKSSSAKVDTDTAFKTIQVKLCYAPISQVDRPWRKTDNKLFKDRSCPHEIVSKAYDK
TPQSLDWTIGLDIPTGTYFVRAYGIDGDGHEVAYGQSTDEGRTTNLFSVHAISGHHVGLDIASTFFSVFS
VVSLFVFFVMEKRKAKLEQRE

>AoNAR2.1
MENQRRNNQEMAASSLFFSPATILGLLLLFSIGSSVEGVLFSKLKQTLIVTASPNHGQVLKAGEDKLTVS
WALNSTVSDSSYKQVKVILCYAPVSQKDRGWRKTVDDLSIDKTCQFDIITKPYTKSKTTFEYMVKRNVPT
ATYFIRAYVLDFSENEVAYGQTTDAAKTTKFFDIIGISGRTASLDIAAGCFSAFSILSLVYFFVKEKRMA
KK

>BvNAR2.2
MGVRGLIIFAAVLCSLVATCYSKGVFADLRNTLTVSASPSGRVDLRAGIDQITVSWRLNRNISNTDSATY
SKVDVKLCYHLESQKDRPWRRTEEDLSRDKTCQFSMVKKDYNSSTDSVTYTVKKNIPTAHYFVRVYVRNA
DNREIAYGQTNGLDLNIKGISGRSTSIDVAASVFSGFSVLSLAFFFFLEKRKAKKLTS

>BrNAR2.1
MCTPPFISNIKSQQTKAYSSSLSKYSDSRISMAIHKSLFASLLICLLFQISHGATKERLFSDLEKGALEV
TAKPSREGVLDAGIDKLSITWKLSSTATKEAEFTTIKVKLCYAPVSQVDRPWRKTENELFKDKSCPHKII
TRAYDKSPQSFEYTLERDIPTGTYFVRAYAVDAKDHEVAFGQSTNEAKSTNLFSVQAISGRHKSLDIASV
CFSVFSVLALLVFFVNEKRKAKIEQSK

>CchNAR2.2
MASTTAIFLATLVICCSLSSSHAEILFSSLKKSLEVTVKHRAGVLMAGEDTLEIDWFLNKTFPAGTDSAY
KTIKLKLCYAPISQKDRAWRKTEDHLKKDKTCLFEIDSTPYKSSNNKFNWTIERDVPTGTFFVRAYILNG
DGHEIGYGQNTDDKKVNNLFDIQAISGRHATLDICSVVFSVFAVVSLFGFFYMEKRNAKASK

>CqNAR2.2
MAVRGLIIFSVLLSSLVASCYGTGLFKDLSNTLTVSTTPTGKVNLKAGKDQITVTWGLNRNVSKLDTSAY
KQVEVKLCFLAESQVDRPWRKTEDELARDKTCQFLVVKKDFTSSSDSFTYTVKKDVPTAHYFVRVYVRDG
PDGKQLAYGQTTGLDLSVKSISGRSASIDIAASIFSAFSVLSLAFFFYLEKKKARRAT

>EsNAR2.1
MAIHKILFASLFICSLIQSSHGATKERLFSDLEKGAFEVTTKPSREGEGVVLDAGIDKLSITWKLNSTAK
EAEFKTIKVKLCYAPISQVDRPWRKTENELFKDKSCPHKIVARPYDSSVKTAQTIDYTLERDIPTGTYFV
RAYAVDAHDHEVAFGQSTDEDKKTNLFSVQAISGRHKSLDIASICFSVFSVLALLVFFVNEKRKAKIEQS
K

>EsNAR2.2
MAIHSFLFISLLLILSSLESSSGTTKDRLFFTNLQNTLEVTAKPIRDGVLESGKDIIRVTWKLKSSVKIDV
DAAIKTVDVRLCYAPISQVDRPWRQSHNELFKDKTCPYKIVSKPYDKISQSLNWIIERDIPTGTYFVRVY
GIDANGHEVAYGQXSNAEKTTNLFSVEAIDGRHISLDAASICFSIFSVVALLVFFMRERRRNPI

>GmNAR2.1
MAAQGPVVVASLLIFCLAGSCYGKVHFSSLKRTLDVTASPKQEQVLEAGLDKITVTWALNKTLPAGTDSA
YKTIKLKLCYAPISQKDRAWRKTEDELKRDKTCQHKIVAKPYDASNKTVQRFEWLVERDVPKATYFVRAY
AFDSNDEEVAYGQTTDAKKSTNLFEINAVSGRHASLDICSICFSAFSVVSLFVFFYIEKRKGKASSSK

>HvNAR2.1
MARSELVMVLLVVVLAAGCCTSAGAVAYLSKLPVTLDVTASPSPGQVLHAGEDVITVTWALNTTQAGKDA
DYKNVKVSLCYAPVSQKEREWRKTHDDLKKDKTCQFKVTQQAYPGAGKVEYRVALDIPTATYYVRAYALD
ASGTQVAYGQTAPTAAFNVVSITGVTTSIKVAAGVFSAFSVASLAFFFFIEKRKKNN

>HvNAR2.2
MARSDLVMALLVAVLAAGCCASAGAVAYLSKLTVTLDVTASPTPGQVLHAGEDVITVTWALNATRPAGDD
AAYKSVKVSLCYAPASQKEREWRKTHDDLKKDKTCQFKVTQQPYAAGAAGGRVEYRVALDIPTAAYYVRA
YALDASGTQVAYGQTAPATAFDVVSITGVTTSIKVAAGVFSTFSVVSLAFFFFIEKRKKNN

>IpNAR2.1
MGDLFSIPVLFFLLFLLVGTAAGEGVLLSTLPRTLIVTASPKQGQVLKAGEDHITVSWGLNKSLSASTDD
AYKNVKVMLCYAPVSQTDRGWRKTNDDLKKDKTCQFEVTVQPYAKSNSSFEYKIKRDVPSATYFLRAYLL
DSSDTKVAYGQTTDAHKSTNLFEIVGISGRSASLDVAAGCFSAFSVVSLLFFFVIEKRKAKKSLN

>MtNAR2.1
MAAHKLVVASLLLCCLAEICYGKDLFSSLKRTIDVTASPKQGQVLLSGVDKISGTWALNKTFPAGTDSSY
KTIKLKLCYAPISQKDRAWRKTEDELSRDKTCQHKMLAMPYNASNKTVQTFEWLIQRDVPQATYFVRAYA
FDSNDKEVAYGQTTNAGKSTNLFEINAISGRHATLDICSVVFSAFSVVSLGVFFYIEKRKGKSPKQ

>OsNAR2.1
MARLAGVAALSLVLVLLGAGVPRPAAAAAAKTQVFLSKLPKALVVGVSPKHGEVVHAGENTVTVTWSLNT
SEPAGADAAFKSVKVKLCYAPASRTDRGWRKASDDLHKDKACQFKVTVQPYAAGAGRFDYVVARDIPTAS
YFVRAYAVDASGTEVAYGQSSPDAAFDVAGITGIHASLKVAAGVFSTFSIAALAFFFVVEKRKKDK

>OsNAR2.2
MARFGAVIHRVFLPLLLLLVVLGACHVTPAAAAAGARLSALAKALVVEASPRAGQVLHAGEDAITVTWSL
NATAAAAAAGADAGYKAVKVTLCYAPASQVGRGWRKAHDDLSKDKACQFKIAQQPYDGAGKFEYTVARDV
PTASYYVRAYALDASGARVAYGETAPSASFAVAGITGVTASIEVAAGVLSAFSVAALAVFLVLENKKKNK

>PfNAR2
MAIQGFLVASILISCFVATCYGVTFSSLQNTLVVTSSPHSDQELKAGEDKITVTWSLNTTFPSGTDSTYK
MVKVKLCYAPISQKDRGWRKTVDNLKKDKTCQHKIVAKPYTASNNTFTYTVQRDVPTATYFVRAYVVNAA
DEEVAFGQSTDSHKATNLFKIQAISGRHISLDIASVCFSAFSIVSLFGFFYMEKRKAK

>PjNAR2.1
MANHVSVIALISLSCLVATCYGNVLFSTLNPTLIVTASPSPPGQVLKAGEDTITVTWSYNSSFPSSTDSD
YRTVKIMLCYAPESQQDRAWRKTVDNLKKDKTCQHKIVSRPYSRSGNNTFTYTVQRDIPTATYFVRAYAL
NAHDTQLAYGQTTNAQKTTNLFGIQAITGRHVSLDIASICFSGFSILSLFGFFYMEKRKAKSQSK

>ShNAR2.1
MAINRVIVATILLSCLAATCYAITFSSLQRTLDLDSSVQAGQVLKAGEDKITVSWKVNATYPAGTESSYK
TVKVLLCYAPVSQKDRGWRKTVDLLKKDKTCQHTIVERAYSASDNSVTYTVKRDVPTATYFIRAYVYNAA
GEEVGYGQTTNADKSANLFAVEAVTGRHVSLDIASACFSAFSVVSLFGFFFMEKRKAKASN

>SiNAR2.1
MAIQGSIVASILLSYLVAACYGATFSSLQNTLVVTASPQAGQVLKGGEDNITVTWSLNNTFPAGTDSDYK

TVKIKLCYAPISQQDRAWRKTEEELKRDKTCQHKIVAKPYSPSTNTFTWTVQRDVPTATYFIRAYVHNSA
DKEVAFGQTTDSHKATNLFEVQAISGRHVSLDIASVCFSAFSVVALFGFFFLEKRKAKASQKS

>SpNAR2.2
MAISTRAIFVATIVICSFLAVCEGEILFSTLKKSLEVKADHKAGVLMAGEDALTLKWSLNNTLPAGSDSG
YKSVKLELCYAPISQKDRAWRKTEDHLKKDKTCQFKIATVPYKSSNNNYNWTIKRDIPTATYFVRAYVLN
GNGEEIGYGQNTDDKKVDNLFSIQAISGRHATLDICSVVFSAFSVVSLFAFFYMEKRKARAGK

>SoNAR2.2
MAVRGLIIFVALLSSLAASCYGVMFHELAHSLTVSSTPSGQANVKAGKDQITVTWALNRSISGVDTSIYR
EVEAKLCYHLESQKDRPWRKTEEEMARDKTCQFAIVKRPYTSSSDSVTYTIKKDVPTAHYFIRVYVRDGP
GGKLIAYGQTTGLDLFVAGISGRSASIDIAASIFSAFSVISLGFFFYLEKKKSKRAT

>SaNAR2.2
MAVRGLIIFVALLSSFVVACYGEGLFSELKQTLTVSASPTGKVSLIAGKDEITVTWGLNSTSTKTTNYKN
VEVKLCYLEESQKDRPWRRTEDDLKRDKTCQFLVAEKEFTTKDDTVTYQVKKDVPSAHYFIRAYVTDGPK
GKLIAYGQTTGLDLKVKGISGRSLAIDVAASVFSGFSVLSLVFFYFMEKSKAKRAS

>SfNAR2.2
MAVRGLIIFVALLSSFVVACYGEGLFSELKQTLKVSSSPTGKVFLMAGKDEITVTWGLNSTSTKTTNYKNVEVKLCY
LEESQKDRPWRKTEDDLKRDKTCQFLVAEKEFTTKDDTVTYKVKKDVPSAHYFIRAYVSDGPKGKLIAYGQTTGLDL
QVKGISGRSLAIDVAASVFSGFSVLSLVYFYFMEKSKAKRAS

>SgNAR2.2
MAIRGLIIFGALLSSFVVACYGEGLFSDLKQTLSVSASPTGKVFLTAGKDEITVTWGLNSTSTKTTNYKNVDVKLCY
LAESQKDRPWRRTEDDLKRDKTCQFLVAEKEFNTKGDKVTYKVKKDVPSAHYFIRAYVKDGPKGKLIAYGQTTGLDI
EVNGISGRSLAIDVAASVFSGFSVLSLVFFYFMEKRKAKRAS

>TaNAR2.2
MAQSKLVMALLVAVLAAGCCASAGAVAYLSKLPVTLDVIASPSPGQVLHAGEDVITVTWALNASRPAGDD
AAYKNVKVSLCYAPASQKEREWRKTHDDLKKDKTCQFKVAQQPYAGAGGRVEYRVALDIPTATYYVRAYA
LDASGTQVAYGQTAPAAAFNVVSITGVTTSIKVAAGVFSTFSVVSLAFFFFIEKRKKNN

>TaNAR2.1
MARQGMVTALLLVVLAAGCCASAGAVAYLSKLPVTLDVTASPSPGQVLHAGEDVITVTWALNASQPAGKD
VDYKNVKVSLCYAPVSQKEREWRKTHDDLKKDKTCQFKVTQQAYPGTGKVEYRVALDIPTATYYVRAYAL
DASGTQVAYGQTAPSSAFNVVSITGVTTSIKVAAGVFSAFSVASLAFFFFIEKRKKNN

>VrNAR2.1
MAAHRLVVVSLLIFCFAGSCYGKVHFSSLKKTLDVTASPKQGQVLEAGTDKITVTWALNNTLPTGTDSAY
KTIKVKLCYAPISQKDRAWRKTEDELSRDKTCQHKIVAKPYDASNRTVQRFEWVIERDVPKATYFVRAYA
FDSNGEEVAYGQTTDAKKSSNLFEINSISGRHASLDICSVCFSAFSVVALFVFFYIEKRKAKASSSSK

> ZosmaNAR2.1
MYSSFLSVAVSVAVFAVVCCVSPLVDAEVMFSSLPENLIVDASPIQGQVLKAGEDNITVSWMLKTNLAPG
VDAKYQSIKIKLCYGPSSQVDRGWRKTRDNLKKDKTCQFNVGESLIYNSNAAQMEQRVTYTISREVPTAM
FFVRAYVLDSNGKKIAYGQSTNAEKNTNLFEVEGITGRNTSLNIAATCFSAFSLMSMTGFYIADRRRKNK
